# pARIS-htt: an optimised expression platform to study huntingtin reveals functional domains required for vesicular trafficking

**DOI:** 10.1186/1756-6606-3-17

**Published:** 2010-06-01

**Authors:** Raúl Pardo, Maria Molina-Calavita, Ghislaine Poizat, Guy Keryer, Sandrine Humbert, Frédéric Saudou

**Affiliations:** 1 Institut Curie, F-91405 Orsay, France; 2 Centre National de la Recherche Scientifique, Unité Mixte de Recherche 3306, F-91405 Orsay, France; 3 Institut National de la Santé et de la Recherche Médicale, Unité U1005, F-91405 Orsay, France

## Abstract

**Background:**

Huntingtin (htt) is a multi-domain protein of 350 kDa that is mutated in Huntington's disease (HD) but whose function is yet to be fully understood. This absence of information is due in part to the difficulty of manipulating large DNA fragments by using conventional molecular cloning techniques. Consequently, few studies have addressed the cellular function(s) of full-length htt and its dysfunction(s) associated with the disease.

**Results:**

We describe a flexible synthetic vector encoding full-length htt called pARIS-htt (**A**daptable, **R**NAi **I**nsensitive &**S**ynthetic). It includes synthetic cDNA coding for full-length human htt modified so that: 1) it is improved for codon usage, 2) it is insensitive to four different siRNAs allowing gene replacement studies, 3) it contains unique restriction sites (URSs) dispersed throughout the entire sequence without modifying the translated amino acid sequence, 4) it contains multiple cloning sites at the N and C-ter ends and 5) it is Gateway compatible. These modifications facilitate mutagenesis, tagging and cloning into diverse expression plasmids. Htt regulates dynein/dynactin-dependent trafficking of vesicles, such as brain-derived neurotrophic factor (BDNF)-containing vesicles, and of organelles, including reforming and maintenance of the Golgi near the cell centre. We used tests of these trafficking functions to validate various pARIS-htt constructs. We demonstrated, after silencing of endogenous htt, that full-length htt expressed from pARIS-htt rescues Golgi apparatus reformation following reversible microtubule disruption. A mutant form of htt that contains a 100Q expansion and a htt form devoid of either HAP1 or dynein interaction domains are both unable to rescue loss of endogenous htt. These mutants have also an impaired capacity to promote BDNF vesicular trafficking in neuronal cells.

**Conclusion:**

We report the validation of a synthetic gene encoding full-length htt protein that will facilitate analyses of its structure/function. This may help provide relevant information about the cellular dysfunctions operating during the disease. As proof of principle, we show that either polyQ expansion or deletion of key interacting domains within full-length htt protein impairs its function in transport indicating that HD mutation induces defects on intrinsic properties of the protein and further demonstrating the importance of studying htt in its full-length context.

## Background

Huntingtin (htt) is a protein of 350 kDa that when mutated causes Huntington's disease (HD). HD is a devastating inherited neurodegenerative disorder characterized by the selective dysfunction and death of particular neurons in the brain [1,2]. The causative mutation is an abnormally expanded CAG tract in the 5'coding region of the htt gene that is translated into a long polyglutamine (polyQ) stretch in the N-terminal part of the protein. HD occurs when there are more than the threshold of 36 glutamines. The mechanisms leading to disease are not fully understood but involve both the gain of new toxic functions and the loss of normal htt function(s) [1-3]. For example, loss of htt function in the transcription of brain-derived neurotrophic factor (BDNF) and in its microtubule (MT)-dependent transport participates in HD pathogenesis [4,5]. We and others have contributed to the identification and characterization of postranslational modifications within htt that regulate the function(s) of both the wild-type protein and the toxicity induced by the mutant version. These findings demonstrate the importance of the protein context. The first identified modification of htt was its phosphorylation at serine 421 (S421). Htt S421 is phosphorylated by Akt and the *Serum and Glucocorticoid-induced kinase *(SGK) and is dephosphorylated by calcineurin [6-9]. Phosphorylation at S421 is abnormally low in disease [9-11]. Dephosphorylation of S421 is associated with reduced htt function in the MT-dependent transport of BDNF in neurons and may contribute to the selective neurodegeneration in cases of HD [12,13]. Htt is cleaved by several proteases, including caspase 6 which may play a crucial role and modify disease progression [14]. PolyQ-htt susceptibility to cleavage is regulated by phosphorylation of serine 434 by Cdk5 [15] and of serine 536 by an unidentified kinase [16]. Also, the specific acetylation of mutant htt at lysine 444 leads to its selective degradation by autophagy, thereby reducing toxicity [17]. Subcellular trafficking of htt and its association with lipid membranes can be modified by palmitoylation of cysteine 214 [18]. Palmitoylation-resistant mutants accelerate formation of inclusions and neuronal toxicity. Mass spectrometry experiments have identified additional phosphorylation sites in the central and carboxy-terminal parts of the protein [16] which may be involved in additional mechanisms regulating its cellular functions. Sequence analysis revealed at least 36 HEAT (**h**untingtin, **e**longation factor 3, PR65/A subunit of protein phosphatase 2**A **and m**T**or) repeats dispersed throughout the protein [19,20]. The presence of these domains and the predicted structure of htt are consistent with a cellular role as a scaffold protein [21,22]. In agreement, more than one hundred interactors have been reported in yeast-two-hybrid screens using various htt fragments as baits [23,24]. The protein sequence, including the central and carboxy-terminal part of the protein, has been very highly conserved throughout evolution. These various observations all indicate the importance of the full-length protein context when addressing htt functional studies. They have also led to the emerging notion that understanding normal htt function is essential if we are going to understand the pathogenic and regulatory events that occur during disease progression in HD patients.

Various cellular and molecular biology techniques can be used to study the function(s) of a particular protein and its dysfunction(s) when mutated. Many of these techniques require cloning the gene of interest into appropriate vector(s) for subsequent characterization. This step can be extremely laborious and time consuming especially when dealing with large proteins, like htt, and may constitute a difficult technical obstacle for extensive functional and genetic analyses. Because of this problem, and despite the very large number of publications concerning HD since the cloning of the htt gene in 1993, most studies have used only short N-terminal fragments of the htt protein and focused on the gain of toxic function elicited by the polyQ stretch. Indeed, expression of short N-terminal fragments (containing the pathogenic expansion), for example the 89 amino acid fragment corresponding to the exon 1, are sufficient to generate a neurological phenotype in mice and to induce the death of various cell types [25]. Although these models reproduce some pathological features observed in HD patients, exon 1 encodes less than 3% of the full-length htt protein; consequently, such studies do not necessarily provide a complete image of the function(s) of the protein and the dysfunction(s) operating during HD. In particular, the translational product of exon 1 does not contain important sites of post-translational modifications, notably S421, L444 and C214, that critically regulate mutant htt toxicity. In addition, events such as caspase 6 proteolysis of the full-length protein are bypassed in such models. Also, functional studies on the role of htt in MT-dependent transport have shown that wild-type full-length htt, but not short amino-terminal fragments such as that encoded by exon 1, stimulates the transport of BDNF-containing vesicles [5]. These observations make clear the need for appropriate tools to study wild-type and pathogenic htt in its full-length protein context.

We report the construction and validation of a complete synthetic htt gene with wild-type and mutant versions. We demonstrate that the wild-type htt encoded by this gene has a positive effect in regulating the trafficking of vesicles and organelles; the pathogenic mutant htt does not. We exploited the versatility of this synthetic htt gene to generate internal deletions and demonstrate that the regions interacting with HAP1 and dynein are required to mediate htt function in cellular trafficking. Thus, this fully synthetic construct will be useful for investigations of htt function and the pathogenic mechanisms underlying HD.

## Results

### pARIS-htt, a synthetic cDNA encoding a tagged full-length version of human huntingtin

**Figure 1:**
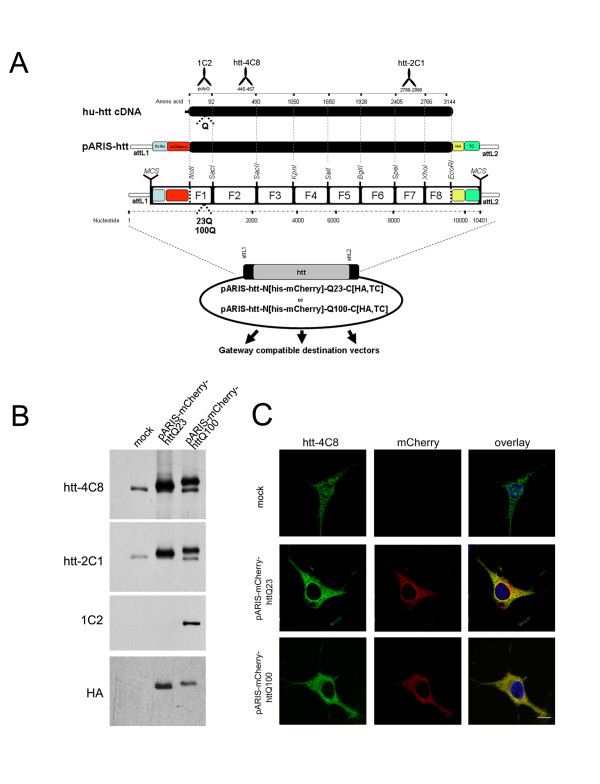
**pARIS-htt an Adaptable, RNAi Insensitive & Synthetic construct encoding human huntingtin**. A) Schematic representation of pARIS-htt. The entire coding sequence is divided into 8 different fragments, each fragment being flanked by unique restriction sites every 1-1.5 kbp and cloned independently into a modified pUC19 backbone. A multi-cassette full-length htt plasmid (pARIS-htt) was generated by assembly of these 8 individual fragments. pARIS-htt construct was tagged with 6×His followed by a mCherry on the amino terminus. The carboxy-terminal part contains HA and tetracysteine (TC) tags. The synthetic construct is fully compatible with the Gateway technology thanks to the introduction of flanking attL sites. (B) pARIS-htt triggers the expression of full-length htt in HEK cells. Cells mock transfected and transfected with pARIS-mCherry-httQ23 or pARIS-mCherry-httQ100 were analyzed by western blot using antibodies raised against different regions of htt: the amino-terminal part (htt-4C8), the carboxy-terminal part (htt-2C1) or the pathogenic polyQ stretch (1C2). The exact epitopes for these antibodies are illustrated in (A). Expression of the different constructs was detected using a high affinity anti-HA antibody. (C) Confocal images of Cos7 mock transfected cells and cells transiently transfected with pARIS-mCherry-httQ23 or pARIS-mCherry-httQ100 constructs. Expression of pARIS-htt is detected as a cytosolic mCherry fluorescent signal which codistributes with htt-4C8 antibody staining. Scale bar 10 μm.

We designed a synthetic cDNA covering the entire sequence of human htt as part of a modular and versatile plasmid expression platform to study the function(s) of the protein. This system includes all the benefits of the Gateway system from Invitrogen. We named this platform pARIS-htt (**A**daptable, **R**NAi **I**nsensitive &**S**ynthetic). The sequence of human htt (GenBank access NM_002111) was optimized for eukaryotic codon usage. We also exploited the degenerate nature of the genetic code to eliminate various restriction sites from the sequence and introduce others, resulting in a synthetic DNA sequence with unique restriction sites every 1-1.5 kbp on average without modifying the amino acid sequence encoded (GenBank access NP_002102)(Figure 1A). The overall construction strategy required first the synthesis and cloning of eight fragments separately in the vector pUC19. The full-length version of the htt gene was then generated by assembling the eight fragments (Figure 1A). In the resulting construct, the entire sequence can be divided into eight different fragments, each single fragment being flanked by unique restriction sites.

The multi-cassette nature of pARIS-htt is advantageous. Any desired mutation of the full-length context can be generated in two steps. The first step is the introduction of the desired mutation into the corresponding 1 to 1.5 kbp fragment in pUC19. The second step is to reintroduce the mutated fragment into the full-length htt cDNA by conventional restriction enzyme digestion and ligation. PolyQ expansion in the htt protein causes HD, so we produced pARIS-htt versions with and without a sequence encoding an abnormal polyQ stretch. We generated a pARIS-htt version encoding a pathogenic polyQ expansion of 100Q by introducing alternate CAG-CAA repeats, because the alternating codons are genetically more stable [26]. The NotI site upstream from the sequence encoding the first N-ter 17 amino acids and the SacI site downstream from that encoding the poly-proline stretch (amino acid position 92) allow the introduction of a sequence encoding a synthetic N-terminal fragment with CAG repeats of any desired size by simple digestion with NotI/SacI followed by insertion of a compatible cassette coding for the polyQ sequence of desired length. We generated two different cassettes coding for 23 and 100Q.

To facilitate functional studies, the pARIS-htt constructs were designed to allow production of various fusion proteins: tagging of the amino terminus of the encoded protein with a 6× histidine-tag to allow protein purification on Ni^2+ ^columns, and fusion to the mCherry protein; the C-terminus was tagged with haemaglutinin (HA) that can be used for immunoprecipitation or immunohistochemistry, and a tetracysteine tag (TC-Tag) that allows the fusion protein to be specifically recognized in living cells by biarsenic labelling reagents such as FlAsH-EDT_2 _and ReAsH-EDT_2 _[27,28]. The sequences encoding the tags can be easily removed by using the unique NotI and EcoRI restriction sites and multicloning sites (MCS) at the two extremities (Figure 1A; sequences and vector maps are provided in additional files 1 and 2). Another important feature of pARIS-htt is its full compatibility with the Gateway technology, which allows recombination-based cloning and all its benefits in terms of time and simplicity. This property is provided by the inclusion of the specific recombination sites attB1 and attB2 flanking the htt coding sequence. First, the htt coding wild-type and mutant sequences were transferred to a general donor plasmid (pDONR201, Invitrogen) to generate Entry vectors in which pARIS-htt is flanked by attL sequences (BP clonase reaction). These Entry vectors are referred as follows: pARIS-htt-N[His-mCherry]Q23-C[HA-TC] and pARIS-htt-N[His-mCherry]Q100-C[HA-TC]. pARIS-htt sequences can then be transferred to the desired destination vector though a second recombination (LR clonase reaction) to generate an expression clone. Numerous commercially available destination vectors can be used for straightforward expression of synthetic human htt protein in diverse biological systems (transient expression in mammalian cells, baculovirus-mediated infection of insect cells, lentiviral-mediated delivery to neural cells or expression in *Drosophila melanogaster*).

### pARIS-htt drives expression of full-length huntingtin and is insensitive to siRNAs targeting the endogenous protein

To validate pARIS-htt constructs, we first generated a pcDNA-based (pcDNA3.2-DEST, Invitrogen) expression vector to drive pARIS-htt expression in mammalian cells. The constructs pARIS-htt^pcDNA3.2^-N[His-mCherry]Q23-C[HA-TC] (pARIS-mCherry-httQ23) and pARIS-htt^pcDNA3.2^-N[His-mCherry]Q100-C[HA-TC] (pARIS-mCherry-httQ100) were used to transfect HEK cells and protein expression was analyzed by immunoblotting with various htt-specific antibodies recognizing different epitopes within the protein, including htt-4C8 whose epitope maps to positions 443-457 of the amino acid sequence and the htt-2C1 antibody raised against the carboxy-terminal region (2788-2990) [29,30] (Figure 1A). These two antibodies gave strong signals for the synthetic htt constructs with migration shifts due to the presence of the tags for pARIS-mCherry-httQ23 and that of the expanded polyQ stretch for pARIS-mCherry-httQ100 (Figure 1B). pARIS-mCherry-httQ100 but not Q23 was selectively recognized by antibody 1C2 that binds specifically to pathogenic polyQ expansions [31]. The synthetic constructs containing a C-terminal HA tag were also efficiently recognized by anti-HA antibodies. We next investigated whether expression of pARIS-htt in transfected Cos7 cells could be detected by direct immunofluorescence due to its N-terminal mCherry fluorescent tag. The fluorescent signals corresponding to pARIS-mCherry-httQ23 and pARIS-mCherry-httQ100 were mainly cytosolic and colocalized with htt-4C8 staining (Figure 1C). This localization is in agreement with previous studies [29].

**Figure 2:**
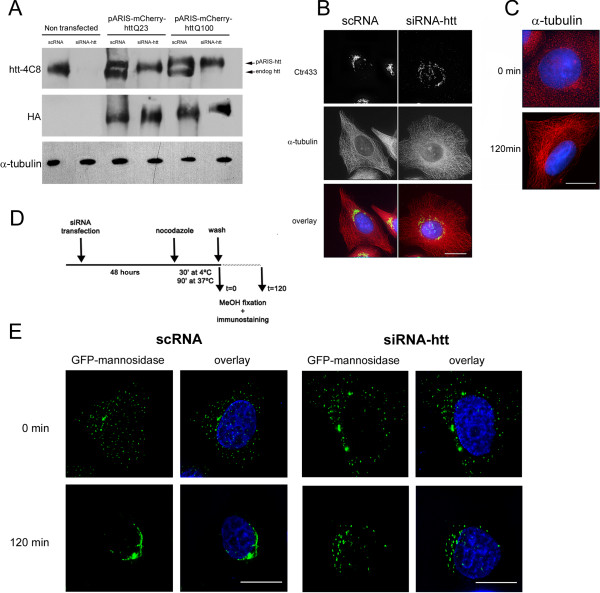
**Huntingtin depletion impairs Golgi reformation after microtubule disruption**. A) HeLa cells were sequentially transfected with scRNA or siRNA-htt and pARIS-mCherry-httQ23/Q100 and finally analyzed by western blot using antibodies that recognize either endogenous and exogenous htt (htt-4C8) or only exogenous htt (HA). Treatment with siRNA-htt (second lane) results in the complete silencing of endogenous htt. Compared to endogenous htt, pARIS-htt displays lower mobility due to fusion with tags. Note that expression levels of pARIS-mCherry-httQ23/Q100 are not modified by siRNA-htt treatment (lanes 4 and 6). α-tubulin is used as a protein loading control. (B) HeLa cells were transfected with scRNA or siRNA-htt, fixed and processed for staining of a Golgi marker (Ctr 433) and α-tubulin. Unlike scRNA-treated cells, cells silenced for endogenous htt display a dispersed Golgi phenotype but an intact MT network. (C) α-tubulin staining before and after nocodazole (NZ) treatment reveals that MT network is entirely reformed 120 min after NZ removal in HeLa cells. (D) A schematic description of the transfection protocol is summarized. (E) HeLa cells stably expressing GFP-mannosidase II were transfected with scRNA or siRNA-htt and treated with NZ for 120 min to allow a complete MT depolymerization. Golgi reformation was monitored 120 min after NZ washout. In scRNA-treated cells the GA becomes again centrally organized. However, cells depleted from endogenous htt still present a dispersed GA at the same time point. Scale bars 10 μm.

The pARIS constructs are suitable for use in gene replacement strategies. Indeed, the sequence was modified to make it insensitive to various siRNAs that efficiently silence human, rat or mouse htt (see Methods section for the list of siRNAs that can be used and additional file 1 for their positions within htt sequence). We previously reported a similar strategy for replacing endogenous htt with exogenous N-ter amino acid fragments of htt [9,13]. Here, we significantly improved the pertinence of this approach, because pARIS-htt allows the re-expression of full-length htt versions in various cellular contexts (human, mouse or rat cells) in which the endogenous htt is silenced. As a proof of principle, we specifically silenced endogenous htt in HeLa cells using a human specific siRNA, siRNA-hu-htt-585, which is particularly effective for knocking-down htt expression in cells of human origin (data not shown). HeLa cells were transfected with control RNA (scRNA) or siRNA-hu-htt-585 using lipofectamine, transfected 24 h later with pARIS-htt constructs, and then incubated for an additional 24 h. Expression of both endogenous and exogenous htt was analyzed by Western blotting (Figure 2A). Production of endogenous htt was completely abolished by the siRNA without affecting pARIS-mCherry-httQ23/Q100, whose expression was detected using an anti-HA antibody. Thus, pARIS-htt is fully insensitive to siRNAs targeting htt and therefore can be used for replacement strategy experiments in cells.

### pARIS-htt can substitute endogenous huntingtin in a Golgi reassembly assay

We exploited this RNAi insensitivity to develop a cellular test and to validate pARIS-htt as a functional htt protein. Htt is found in the Golgi apparatus (GA) [32-34]. In our hands, a significant fraction of pARIS-mCherry-httQ23 was similarly localized in discrete sites in the GA (data not shown). Knock-down of htt in cells results in the disruption of GA structure, leading to the suggestion that htt plays an active role in the maintenance of the GA structure near the cell centre [34]. Studies in which microtubules (MTs) were depolymerized or molecular motors were inactivated indicate that MTs and minus end-directed motors are also required to ensure the structural integrity and the perinuclear localization of the GA [35-40]. The requirement for htt in the organisation and maintenance of the Golgi is linked to the interaction between htt and components of the dynein/dynactin complex [34]. We therefore set up a cellular test to assess htt function in the transport of Golgi-derived vesicles. We used HeLa cells stably expressing GFP-mannosidase II, a key enzyme of N-linked glycan processing often used as a medial Golgi marker [41]. As expected, silencing of htt was associated with the disruption of the GA structure as shown by the dispersion of Ctr433, a marker of the cis/median Golgi [42](Figure 2B). Most cells depleted of endogenous htt displayed spread GA, which was in many cases fully vesicular instead of being organized into compact perinuclear stacks as observed in most control (scRNA-treated) cells (Figure 2B). We next investigated the role of htt in MT-dependent assembly of the GA (Figure 2C-2E). To do so, we treated the cells with nocodazole (NZ, 4 μM, 120 min) to allow complete depolymerization of the MT network (Figure 2C, upper panel) and dispersion of the GA into numerous ministacks (Figure 2E, upper row) that localize at the exit sites of the endoplasmic reticulum [43]. GA integrity was then analyzed 120 min after NZ washout (Figure 2E, lower row). In scRNA-treated cells the Golgi structure became centrally reorganized again, after NZ washout, concomitant with the reformation of the MT network (Figure 2C, lower panel). In most if not all htt-silenced cells, the Golgi remained dispersed 120 min after NZ washout (Figure 2E, lower row) despite the MT network being completely reconstituted (not shown).

**Figure 3:**
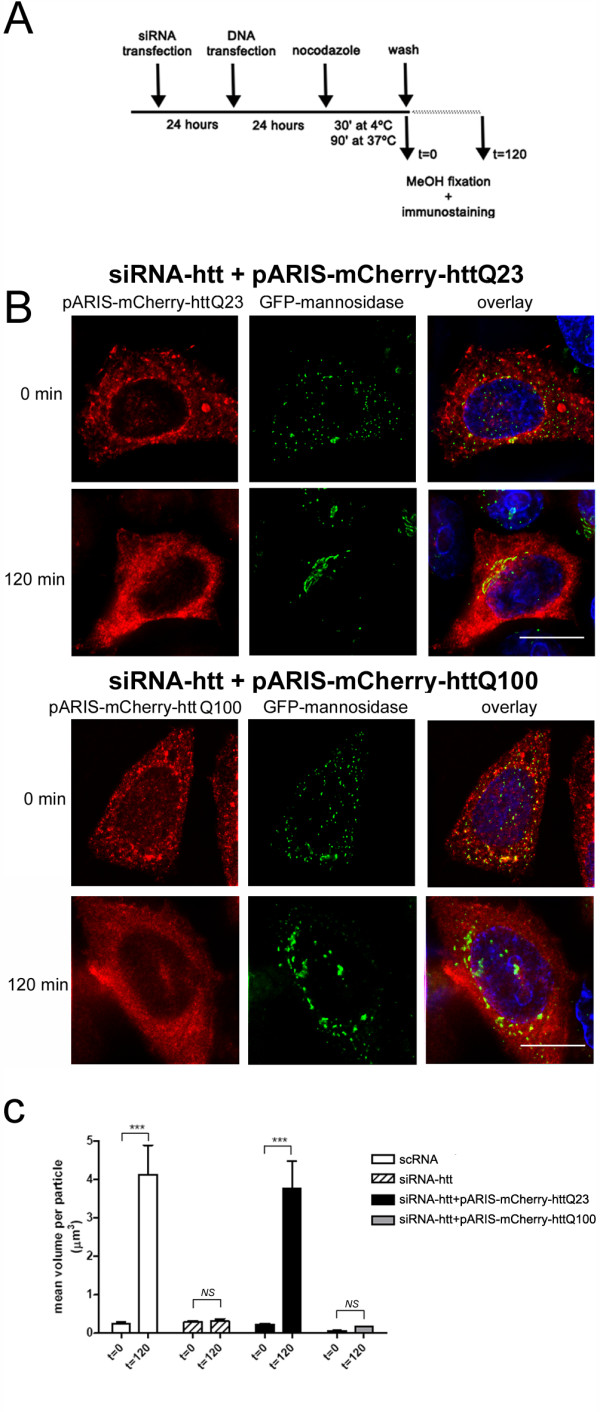
**pARIS-mCherry-httQ23 but not pARIS-mCherry-httQ100 restores Golgi reassembly after endogenous huntingtin depletion**. A) Gene replacement experiments and Golgi reformation assays were performed adding back pARIS-mCherry-httQ23/Q100 in cells depleted from endogenous htt following the protocol indicated in the scheme. (B) Representative images of cells expressing pARIS-mCherry-httQ23 (upper pannel) or pARIS-mCherry-httQ100 (lower panel) at t = 0 and t = 120 after NZ washout. While cells expressing pARIS-mCherry-httQ23 completely reassemble the GA into tight stacks, cells expressing pARIS-mCherry-httQ100 display scattered Golgi fragments that are unable to reassemble in the perinuclear region. (C) Quantification of the GA reassembly is presented as an analysis of mean Golgi particle volume (μm^3^) before and after NZ washout for different treatments. Results were obtained from 3 independent experiments in which 280 cells were analyzed. One way ANOVA followed by Fisher's *Post-hoc *test: ***p < 0.0001; NS non significant. All comparisons are t = 0 vs t = 120; scRNA: 0.283 ± 0.044 vs 4.126 ± 0.771; siRNA-htt 0.073 ± 0.008 vs 0.158 ± 0.06; siRNA-htt + pARIS-mCherry-httQ23: 0.222 ± 0.035 vs 3.763 ± 0.712; siRNA-htt + pARIS-mCherry-httQ100: 0.062 ± 0.006 vs 0.171 ± 0.013.

We next tested whether exogenous expression of pARIS-htt could complement the loss of endogenous htt for MT-dependent assembly of the GA. Expression of endogenous htt was knocked-down by treatment with siRNA-htt. Then, pARIS-mCherry-httQ23, insensitive to the siRNA-htt used, was expressed and cells were treated with NZ for 120 min (Figure 3A). We assessed the reassembly of the GA 120 min after NZ washout. A significant fraction of siRNA-htt-treated cells expressing pARIS-mCherry-httQ23 could efficiently reorganize the GA into stacks in the perinuclear region (Figure 3B). Thus, pARIS-mCherry-httQ23 can substitute for endogenous htt to reassemble the GA into tight stacks.

The role of wild-type htt in GA maintenance has been previously studied [34]. However, it is not known whether pathogenic htt has altered functions in the reassembly of Golgi-derived membranes. We therefore used the same approach but with expression of pARIS-mCherry-httQ100 in cells depleted of endogenous htt. After NZ washout, the highly dispersed GA was unable to reassociate completely into a well-defined perinuclear structure (Figure 3B). Next we developed a system to quantify GA reassembly in this experimental model. We determined the mean volume of Golgi particles before (t = 0) and 120 min after NZ washout (t = 120, figure 3C). NZ treatment induced the complete disintegration of the GA into numerous ministacks with a mean volume of 0.28 ± 0.04 μm^3^. In control cells (scRNA), 120 min after NZ washout, the mean volume per particle increased approximately 15 fold (4.13 ± 0.77 μm^3^, p < 0.0001). In cells depleted of endogenous htt, Golgi-derived vesicles completely failed to cluster and fuse (siRNA-htt, t = 0 vs t = 120; p = 0.634, NS). Expression of pARIS-mCherry-httQ23 in htt-depleted cells completely restored the assembly of Golgi ministacks (3.763 ± 0.71 μm^3^, t = 0 vs t = 120, p < 0.0001) and their transport to perinuclear regions. By contrast, pARIS-mCherry-httQ100 was unable to promote reassembly of the GA (p = 0.432, NS). This experiment indicates that synthetic-Q23 htt restores MT-dependent assembly of the GA in cells with no endogenous htt. More importantly, it shows that the pathogenic polyQ expansion impairs the htt function allowing Golgi reassembly.

### The dynein/dynactin-interacting domains of huntingtin are required for Golgi apparatus reassembly

Our findings indicate that physiological organization of the GA requires wild-type htt and that this function of htt is impaired by polyQ expansion. This is in agreement with previous studies linking htt function to the dynein/dynactin-dependent transport of organelles along MTs [5,12,13,34]. Htt interacts with the dynein intermediate chain (DIC) via a minimal interaction region mapping to amino acid positions 536-698 of htt [34] and with dynactin via HAP1 [44-46] with a minimal interacting region corresponding to amino acids 171-230 of htt [47]. We have previously shown that the htt/HAP1 complex is necessary for vesicle transport along MTs. Depletion of HAP1 through siRNA treatment impairs BDNF transport along MTs in neuronal cells [5]. We have also shown that expression of wild-type exon 1 of htt that lacks the HAP1 binding region, does not stimulate BDNF transport in neuronal cells; by contrast, a 480 amino acid N-ter fragment can stimulate such transport. Although informative, these experiments have limited relevance because, due to the difficulty of manipulating full-length htt, they are based on the expression of truncated forms with most of the protein being deleted. Therefore, it is unknown whether these domains are required within a full-length htt context as mediators of htt regulatory function in the dynein/dynactin complex.

**Figure 4:**
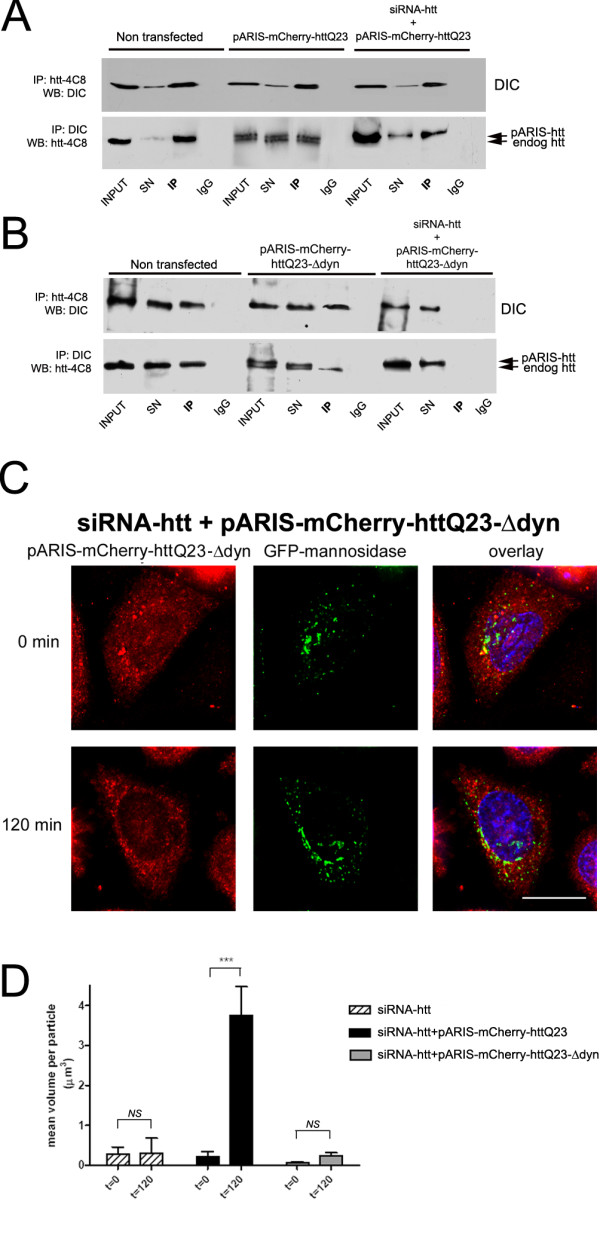
**Htt requires dynein interacting domain to facilitate the transport of Golgi-derived vesicles**. A) HEK cells were treated with scRNA or siRNA-htt prior transfection with pARIS-mCherry-httQ23. Cellular lysates were immunoprecipitated using htt-4C8 or anti-dynein (DIC) antibodies and immunocomplexes were subjected to SDS-PAGE to detect either htt or dynein. Dynein co-precipitates with htt when htt-4C8 antibody is used to pull-down endogenous and exogenous htt (Upper panel). Conversely, immunoprecipitation of dynein (lower panel) pulls down both endogenous and exogenous htt (indicated by arrows, lower mobility band corresponding to pARIS-mCherry-httQ23). The same amount of mouse or rabbit IgG's were used as internal immunoprecipitation controls. SN stands for supernatant; IP denotes immunoprecipitation. B) A deletion mutant lacking the minimal dynein interaction domain, denoted as pARIS-mCherry-httQ23-Δdyn, is unable to bind to endogenous dynein (lane 11). (C) Golgi reassembly was monitored in HeLa cells stably expressing GFP-mannosidase II, silenced for endogenous htt and expressing pARIS-mCherry-httQ23-Δdyn as the only cellular source of htt. Most of the cells expressing pARIS-mCherry-httQ23-Δdyn failed to reassemble the GA after NZ washout, suggesting that htt-dynein interaction is required to transport retrogradely Golgi-derived vesicles. Scale bar 10 μm. (D) Quantification of the Golgi dispersion as the mean volume per Golgi particle (μm^3^) before and after after NZ washout for the different treatments. Results were obtained from 3 independent experiments in which 192 cells were scored. One way ANOVA followed by Fisher's *Post-hoc *test: ***p < 0.0001; NS non significant. All comparisons t = 0 vs t = 120; siRNA-htt 0.073 ± 0.008 vs 0.158 ± 0.06; siRNA-htt + pARIS-mCherry-httQ23: 0.222 ± 0.035 vs 3.763 ± 0.712; siRNA-htt + pARIS-mCherry-httQ23-Δdyn: 0.073 ± 0.010 vs 0.244 ± 0.072.

To investigate the htt-dynein interaction, we generated a version of pARIS-htt from which part of the dynein-interacting region was deleted (pARIS-mCherry-httQ23-Δdyn). We used immunoprecipitation experiments to test the ability of this deletion mutant to bind dynein. We used an antibody raised against htt (htt-4C8) to pull-down both endogenous and synthetic htt from non transfected cells and from cells expressing pARIS-mCherry-httQ23 (Figure 4A, upper panel). Under these conditions, dynein co-immunoprecipitated with htt provided the cells express either endogenous htt or pARIS-mCherry-httQ23. Similarly, both endogenous and exogenous htt were co-immunoprecipitated with dynein as shown by the presence of a doublet band (Figure 4A, lower panel). This doublet band was observed in cells transfected with pARIS-mCherry-httQ23 but was absent from non transfected cells and from cells expressing pARIS-mCherry-httQ23 but silenced for endogenous htt. Silencing experiments and re-expression of htt siRNA-insensitive constructs were then used to assess the interaction between dynein and the various exogenous htt constructs. In particular, we tested the interaction between dynein and full-length htt lacking the internal dynein-binding domain. Anti-htt antibodies could not immunoprecipitate dynein from pARIS-mCherry-httQ23-Δdyn expressing cells silenced for endogenous htt (Figure 4B, upper panel). Conversely, although endogenous htt was efficiently immunoprecipitated by an anti-dynein antibody, pARIS-mCherry-httQ23-Δdyn was not (Figure 4B, lower panel). This strongly indicates that pARIS-mCherry-httQ23-Δdyn lacking the dynein-interaction domain does not bind dynein.

Next we studied the effect of the expression of pARIS-mCherry-httQ23-Δdyn on GA reassembly. Unlike in cells expressing pARIS-mCherry-httQ23 (Figure 3B, upper panel), GA stacks remained dispersed in cells expressing pARIS-mCherry-httQ23-Δdyn (Figure 4C). We then quantified GA reassembly (as in Figure 3C) and found that the GA clearly failed to reassemble following NZ washout (Figure 4D). This demonstrates a strong defect in the fusion of Golgi-derived mini-stacks in the presence of pARIS-mCherry-httQ23-Δdyn (siRNA-htt + pARIS-mCherry-httQ23-Δdyn, t = 0 vs t = 120; p = 0.4883, NS). In summary, we show that the htt-dynein interaction is required for the positive effect of htt on GA fusion and transport.

Another mechanism by which htt may regulate the dynein-dynactin complex involves the interaction between htt and huntingtin-associated protein 1 (HAP1), the first htt-interacting protein described. The association between these two proteins is enhanced when htt contains a pathogenic polyQ stretch [44]. HAP1 interacts with dynactin [45,46] and plays a key role in MT-dependent transport of organelles in concert with htt [5,12,48]. Since the minimal interacting region of htt for HAP1 has been mapped to amino acid positions 171-230 of htt [47], we generated a version of pARIS-httQ23 deleted for this domain referred to hereafter as pARIS-mCherry-httQ23-ΔHAP1.

**Figure 5:**
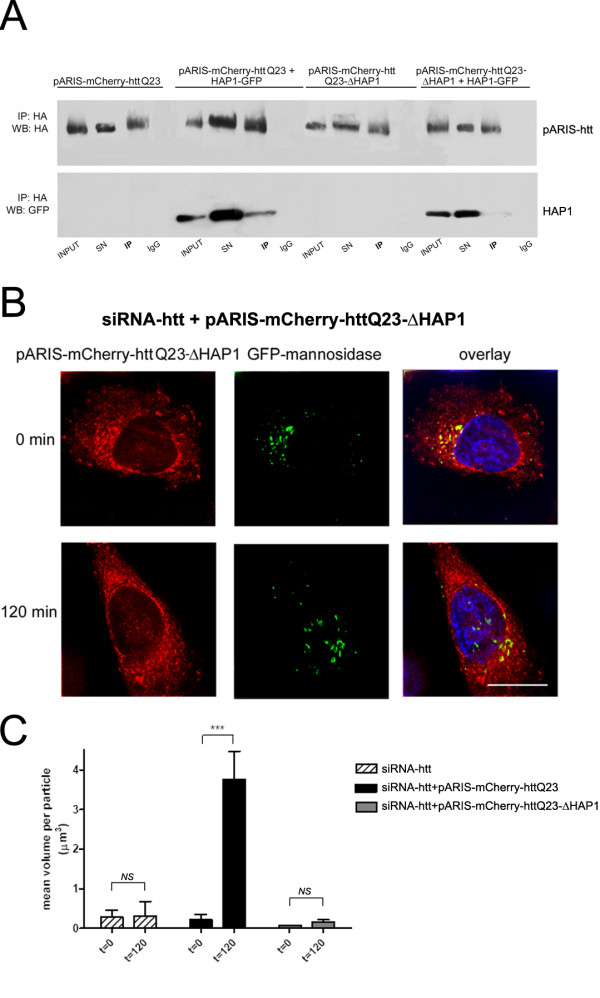
**Htt requires HAP1 interacting domain to facilitate the transport of Golgi-derived vesicles**. A) HEK cells were transfected with pARIS-mCherry-httQ23 or a deletion mutant for the minimal HAP1 interaction domain (denoted as pARIS-mCherry-httQ23-ΔHAP1) in the absence or presence of HAP1-GFP. Exogenous htt was immunoprecipitated (IP) from cell lysates using a HA antibody and immunocomplexes were analyzed for the presence of HAP1-GFP. Immunoprecipitations with mouse IgGs were used as a specificity control. (B) Golgi reformation assays were done in HeLa cells stably expressing GFP-mannosidase II as described previously. Representative image of a pARIS-mCherry-httQ23-ΔHAP1 expressing cell failing to reconstitute the GA after NZ washout. Scale bar 10 μm. (C) Quantification of the Golgi dispersion as the mean volume per Golgi particle (μm^3^) before and after NZ washout for the different treatments. Results were obtained from 3 independent experiments in which 190 cells were scored. One way ANOVA followed by Fisher's *Post Hoc *test: ***p < 0.0001. NS, non significant. All comparisons t = 0 vs t = 120; siRNA-htt 0.073 ± 0.008 vs 0.158 ± 0.06; siRNA-htt + pARIS-mCherry-httQ23: 0.222 ± 0.035 vs 3.763 ± 0.712; siRNA-htt + pARIS-mCherry-httQ23-ΔHAP1: 0.073 ± 0.080 vs 0.159 ± 0.060.

We analyzed whether the pARIS-mCherry-httQ23-ΔHAP1 mutant could bind to HAP1. We co-transfected HEK cells with GFP-tagged HAP1 and either pARIS-mCherry-httQ23 or pARIS-mCherry-httQ23-ΔHAP1. Co-immunoprecipitation experiments demonstrated that the htt construct not containing the 170-268 amino acid region did not bind to HAP1 (Figure 5A) and therefore that this region is necessary for interaction between full-length htt and HAP1. We next tested the effect of this deletion on Golgi reassembly. Unlike cells expressing pARIS-mCherry-httQ23, cells expressing pARIS-mCherry-httQ23-ΔHAP1 could not reorganize the GA even 120 min after NZ washout (Figure 5B), despite complete reorganization of the MT network (not shown). Quantification of the mean volume of Golgi-derived particles (Figure 5C) confirmed the substantial defect in the fusion of GA ministacks in cells expressing pARIS-mCherry-httQ23-ΔHAP1 (t = 0 vs t = 120, p = 0.63, NS). Our results show the importance of HAP1 in the MT-dependent transport of GA-derived vesicles.

Together, our results further extend the role of htt in the maintenance of GA. Indeed, we demonstrate that the capacity of htt to regulate the retrograde transport of dispersed Golgi vesicles to form highly organized stacks around the perinuclear region requires a functional interaction between htt and both dynein and HAP1.

### pARIS-httQ23 but not pARIS-httQ100, pARIS-httQ23-Δdyn nor pARIS-httQ23-ΔHAP1 promotes BDNF transport in neuronal cells

MT-dependent transport of vesicles, such as BDNF, is regulated by the association between htt and the dynein/dynactin complex [5,12,13,34]. Wild-type htt has a positive effect on vesicular dynamics whereas this function is lost in HD [5,12,13,34]. Therefore, the regulatory functions of htt in vesicular trafficking can be evaluated by comparing neurons that express wild-type htt or mutant htt. We previously described the effect of wild-type and pathogenic versions of htt on the dynamics of vesicles that contain eGFP or mCherry-tagged BDNF [5,12,13]. These approaches are sensitive enough to be used to evaluate drugs that restore MT-dependent transport that is altered during HD [5,9,12,13,49].

**Figure 6:**
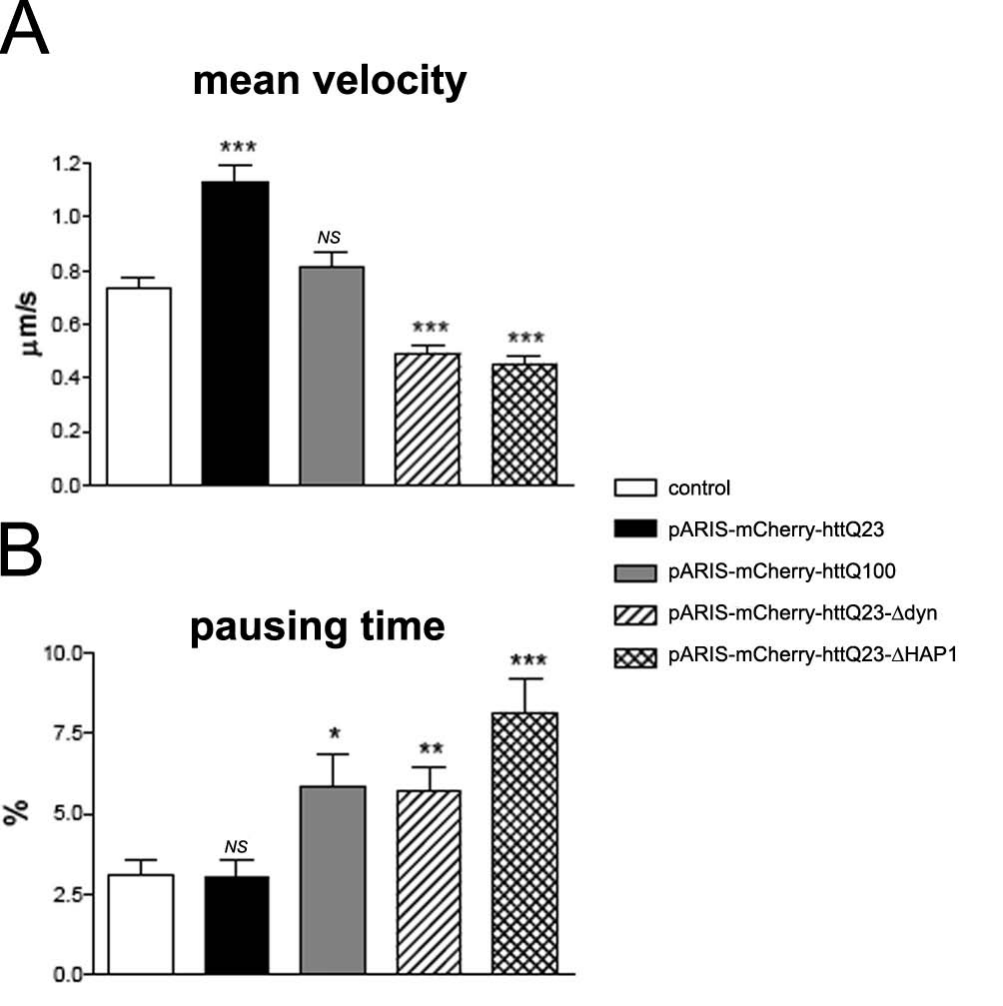
**pARIS-mCherry-httQ23 facilitates BDNF transport through interaction with dynein and HAP1**. A) Fast 3D videomicroscopy was performed to analyze the dynamics of BDNF-eGFP-containing vesicles in mouse neuronal cells expressing BDNF-eGFP alone or cotransfected with pARIS-mCherry-httQ23/Q100 or dynein/HAP1 deletion mutants. Overexpression of pARIS-mCherry-httQ23 recapitulates the transport function of wild-type htt and significantly increases the mean velocity of BDNF-containing vesicles compared to controls values (BDNF transfection alone). Nor the polyQ version neither pARIS-htt deletion mutants are able to stimulate the transport of BDNF containing vesicles. The pausing time of moving vesicles is quantified in (B). Mean overall velocity is indicated as μm/sec. Data were obtained from three independent experiments (control: 4805 tracks from 39 cells; pARIS-mCherry-httQ23: 1970 tracks from 20 cells; pARIS-mCherry-httQ100: 1670 tracks from 18 cells; pARIS-mCherry-httQ23-Δdyn: 4603 tracks from 25 cells; pARIS-mCherry-httQ23-ΔHAP1: 4029 tracks from 20 cells). Fisher's analysis: *P < 0.05; **P < 0.01, NS, non significant.

We analyzed the dynamics of BDNF-eGFP-containing vesicles in mouse neuronal cells using fast 3D videomicroscopy followed by deconvolution. Videomicroscopy was performed one day after electroporation of cells with BDNF-eGFP alone or BDNF-eGFP with pARIS-mCherry-httQ23 and pARIS-mCherry-httQ100, in conditions in which no apparent toxicity was observed. The combination of mCherry-htt and BDNF-eGFP facilitated identification of cells to be recorded during videoexperiments. pARIS-mCherry-httQ23 showed a similar transport function as wild-type htt, significantly increasing the mean velocity of BDNF vesicles (1.132 ± 0.030 μm/s compared to the control value: 0.733 ± 0.044 μm/s; ***p < 0.0001, figure 6A). The presence of pARIS-mCherry-httQ100 did not stimulate BDNF transport (0.810 ± 0.056 μm/s). In cells expressing pARIS-mCherry-httQ100, the pausing time, corresponding to the percentage of time the vesicles spent without moving, was significantly longer than in controls (from 3.09 ± 0.49% in control cells to 5.83 ± 1.03%, figure 6B). The pausing time in cells expressing pARIS-httQ23 was not significantly different from control values (3.00 ± 0.38%).

Htt function in MT-dependent transport of BDNF also involves dynein/dynactin and HAP1. We therefore investigated BDNF dynamics in neuronal cells expressing pARIS-mCherry-httQ23-Δdyn or pARIS-mCherry-httQ23-ΔHAP1. Relative to control values, pARIS-mCherry-httQ23 increased BDNF trafficking in cells, whereas each pARIS-mCherry-httQ23-Δdyn and pARIS-mCherry-httQ23-ΔHAP1 mutants reduced the mean velocity (Figure 6A). Similarly, we also observed a significantly longer pausing time of BDNF-containing vesicles in cells expressing pARIS-mCherry-httQ23-Δdyn or -ΔHAP1 mutants than in cells expressing pARIS-mCherry-httQ23 (Figure 6B). These findings further extend the functional role of full-length htt as a key regulator of MT-dependent transport of organelles in cells. Furthermore, we clearly show that in mammalian neuronal cells, in a physiological full-length protein context, this positive function is mediated by two independent domains of htt protein: the HAP1- and dynein-interacting regions.

## Discussion

There is currently substantial evidence consistent with htt being a scaffold protein required for diverse cellular functions, including various intracellular trafficking processes. Unravelling htt functions in different tissues and how these functions are spatially and temporally tuned to the needs of the cell (via a plethora of post-translational modifications) is extremely complex. In particular, it requires working in the context of the full-length protein. Due in part to htt being a very large protein, this approach has been technically difficult and most experiments addressing htt function, toxicity or post-translational modifications have used short N-terminal fragments of htt; many studies have been based on exon-1 that corresponds to less than 3% of the protein. Here we present a flexible platform to render working with the full-length protein much more straightforward. Our pARIS-htt platform can be used for the construction of tagged and mutant versions of htt in a full-length context. We successfully produced tagged versions of wild-type and mutant htt in various cell lines and generated mutants unable to interact with known protein partners: dynein and HAP1 (pARIS-mCherry-httQ23-Δdyn and pARIS-mCherry-httQ23-ΔHAP1). In the absence of htt, the GA is disrupted, indicating that htt is required to maintain GA organization around the centrosome [33,50]. Indeed, a fraction of htt localizes to the GA and may serve to regulate the post-Golgi trafficking of proteins [32]. We used this property to establish reconstitution experiments to validate our pARIS-htt constructs. Our experimental model is based on the complete silencing of endogenous htt and the expression of various synthetic htt constructs in HeLa cells stably expressing a fluorescent GA-resident protein (GFP-mannosidase II). We used NZ treatment and monitored subsequent reassembly of the GA. In cells expressing pARIS-mCherry-httQ23, the GA was completely reassembled 120 min after NZ washout. By contrast, cells expressing any of the three mutant pARIS-htt constructs (pARIS-mCherry-httQ100, pARIS-mCherry-httQ23-Δdyn or pARIS-mCherry-httQ23-ΔHAP1) failed to reassemble their GA: dispersed ministacks were observed, instead of tightly organized GA around the cell centre, suggesting a defect in the retrograde transport of Golgi-derived vesicles. Quantification of the mean volume of Golgi-derived particles revealed a profound defect in the fusion events in cells expressing any of the mutants, a defect which was not observed in cells expressing pARIS-mCherry-httQ23. These data are in agreement with previous results linking htt function to the maintenance of the GA via dynein [50]. We also validated pARIS-htt constructs for their function in the MT-dependent transport of BDNF-containing vesicles: pARIS-mCherry-httQ23 made a positive contribution to transport whereas this function was lost in neuronal cells expressing pARIS-mCherry-httQ100. Interestingly, BDNF transport, as assessed by measuring BDNF vesicle velocities, was disrupted more strongly by pARIS-mCherry-httQ23-Δdyn and pARIS-mCherry-httQ23-ΔHAP1 than by pARIS-mCherry-httQ100. These experiments demonstrate the requirement of the dynein (633-672) and HAP1 (168-270) interacting regions for htt function in the context of the full-length protein. Furthermore, they suggest that htt regulates vesicular trafficking via distinct but functionally important domains. These results support the notion of htt as a scaffold protein linking vesicles and MTs and promoting the association and regulation of components of the molecular motor machinery, including HAP1 and the motors dynein or kinesin. In agreement with this view, phosphorylation of htt at S421 regulates the recruitment of kinesin-1 to the motor complexes thereby coordinating the directionality of vesicular transport in cells [12]. Moreover, our results strongly suggest that pathogenic polyQ expansions may influence the protein's conformation and its association with motor complexes.

The study of htt function(s) in health and disease is complex, because the protein is widely distributed, but the pathological mutant disables only a small subset of neurons and does so only after many years. Numerous questions concerning the cellular functions of wild-type htt remain unanswered. The role of htt in the regulation of vesicular trafficking is one of its best-described functions [5,12,13,34] and is certainly not limited to its association with dynein or HAP1. Indeed, the contribution of htt to different membrane trafficking events involves other protein partners, such as HIP-1 [51], HAP40 [52], Rab8/optineurin [32,53] or Rab11 [54]. Determination of the true contribution of the reported vesicular trafficking defects to the pathology of HD will certainly require more comprehensive studies. Any such studies would be strengthened by working with the full-length protein, so pARIS-htt constitutes a valuable expression platform for future investigations.

Finally, the combination of yeast-two-hybrid techniques with biochemical approaches led to the identification of more than 100 non redundant htt-interactors. These factors can be classified into different functional groups, including proteins involved in cytoskeletal organization, signal transduction, synaptic transmission, proteolysis and regulation of transcription or translation [23,24,55]. It is important to validate these interacting proteins as *bona fide *genetic modifiers, so that they can then be used to provide insight into the normal function of htt in neuronal and non-neuronal cells, and into the molecular pathogenesis of HD. Here again, pARIS-htt may be a valuable tool for use with other biological approaches for exploring these issues.

## Conclusions

We present a comprehensive set of vectors designed for mutation/tagging and expression of full-length huntingtin. We hope this vector platform will be of value to the scientific community and facilitate functional and genetic studies of htt in the near future.

## Methods

### Statistical analyses

InfoStat software version 2009 (InfoStat Group, FCA, Cordoba National University, Argentina) was used for the analysis of variance and followed by a *post hoc *LSD Fisher's test. Data are expressed as mean +/- S.E.M. *P < 0.05;**P < 0.01;***P < 0.001.

### Constructs and siRNA

The plasmid encoding BDNF-eGFP was previously described [5,56]. BDNF-eGFP shows cellular localization, processing, and secretion properties indistinguishable from those of endogenous BDNF. The plasmid encoding for the huntingtin-associated protein 1 (HAP1) tagged with GFP was a gift of XJ Li (Emory University, Atlanta, USA). The siRNA targeting human huntingtin (siHtt-hu585, Eurogentec, Seraing, Belgium) corresponds to the coding region 279-298 of human htt mRNA (NCBI ref. seq. NM_002111). The control RNA (scRNA, ATCGAGCTACCACGAACGCTT, Eurogentec) has a unique sequence which does not match to any sequence in the genome of interest.

### Construction of pARIS-htt

pARIS-htt was engineered based on the cDNA of full-lengh human htt using OptGene (Ocimum Biosolutions, Hyderabad, India) gene optimizing tool. Gene synthesis was performed by assembly of oligonucleotides using proprietary in-house protocols of BaseClear BV (Leiden, Netherlands). The original sequence was designed with a polyglutamine stretch of 23 glutamines. Glutamine repeats were encoded by alternate CAG/CAA codons to provide more genetic stability. The first base on the start translation codon is considered position number 1.

The pARIS-htt sequence has been rendered insensitive to different siRNAs commonly used in our laboratory: siHtt-1.1 AAGAACTTTCAGCTACCAA (human specific, position 275-293); siHtt-hu585 AACTTTCAGCTACCAAGAAAG (human specific, position 279-298); siHtt-6: AAGCTTTGATGGATTCTAA (human specific, position 474-492); siHtt-13: GCAGCTTGTCCAGGTTTAT (human, rat and mouse specific, position 1062-1080). siHtt-hu585 was used in this study because it is particularly effective to knock-down endogenous htt expression in cells of human origin (referred as siRNA-htt throughout the text).

The full-length engineered pARIS-htt construct was entirely sequenced and inserted into HindIII/BamHI sites of a pUC19 variant (Baseclear BV) for amplification. The pARIS-htt sequence contains flanking attL1 and attL2 sites to allow recombination into pDON201 donor vector (BP clonase reaction, Invitrogen, Carlsbad, USA). A second recombination with pcDNA3.2-DEST (Invitrogen) using LR clonase was necessary to generate a pcDNA3-based destination vector. Recombinations were done in a 10 μl final volume following instructions provided by the manufacturer. Amplification of the constructs was done in TOP10 or DH5α E. coli strains (Invitrogen). A DNA fragment of htt containing a polyQ stretch of 100 glutamines was synthesized using alternative protocols by Geneart AG (Regensburg, Germany) and inserted into NotI/SacI sites of pARIS-htt to replace the 23Q stretch. Vector maps are available in additional file 1.

We use the following nomenclature to describe the first step constructs in the Entry vector: pARIS-htt-N[His-mCherry]Q23-C[HA-TC] and pARIS-htt-N[His-mCherry]Q100-C[HA-TC]. These constructs were transposed to pcDNA3.2 to generate pARIS-htt^pcDNA3.2^-N[His-mCherry]Q23-C[HA-TC] (pARIS-mCherry-httQ23) and pARIS-htt^pcDNA3.2^-N[His-mCherry]Q100-C[HA-TC] (pARIS-mCherry-httQ100).

To generate the htt construct deleted for the dynein-interacting domain, the deletion was first generated within the F3 fragment (pUC19-F3Δ633-672), transposed to Entry-based pARIS-htt by insertion of SacII/KpnI (fragment 3) generating pARIS-htt-N[His-mCherry]Q23-Δ633-672-C[HA-TC] and next transposed to pcDNA3.2 to generate pARIS-htt^pcDNA3.2^-N[His-mCherry]Q23-Δ633-672-C[HA-TC] hereafter denoted pARIS-mCherry-httQ23-Δdyn. To generate the htt construct deleted for the HAP1 binding domain, the deletion was first generated within the F2 fragment (pUC19-F2Δ170-268), transposed to Entry-based pARIS-htt by insertion of SacI/SacII (fragment 2) generating pARIS-htt-N[His-mCherry]Q23-Δ170-268-C[HA-TC] and next transposed to pcDNA3.2 to generate pARIS-htt^pcDNA3.2^-N[His-mCherry]Q23-Δ170-268-C[HA-TC] hereafter denoted pARIS-mCherry-httQ23-ΔHAP1. Requests for constructs may be sent to the following e-mail address: paris-htt.constructs@curie.fr.

### Cell Culture

HEK and Cos7 cells were grown at 37°C in 5% CO2 in Dulbeco's modified Eagle's medium (DMEM) supplemented with 10% bovine calf serum, 1% L-glutamine and antibiotics (50 units/ml penicillin and 50 μg/ml streptomycin). HeLa cells stably expressing GFP-mannosidase II (gift of F. Perez, Institut Curie, Paris, France), were grown at 37°C in 5% CO_2 _and cultured in DMEM supplemented with 10% bovine calf serum, 1% L-glutamine and 400 μg/ml geneticin (Gibco, Carlsbad, USA). Mouse neuronal cells, ST*Hdh*^+/+ ^cells derived from immortalized striatal progenitor cells were grown as previously described [57].

### Cell Transfection

For pARIS-htt expression analysis, HEK cells were transfected with pARIS-mCherry-httQ23, pARIS-mCherry-httQ100 or equivalent amount of empty vector, using the calcium phosphate method [58]. Western blot analysis was performed after 24-48 h.

For immunofluorescence experiments, Cos7 cells seeded in 12-well plates with 18 mm coverslips were transfected with pARIS-mCherry-httQ23, pARIS-mCherry-httQ100 or equivalent amount of empty vector using FuGENE reagent (Roche, Mannheim, Germany) according to the manufacturer's instructions. Immunostaining was done after 48 h.

For gene replacement experiments, HeLa cells stably expressing GFP-mannosidase II were seeded in 12-well plates with 18 mm coverslips. Sequential transfection was performed as following: attached cells were first transfected using Lipofectamine 2000 (Invitrogen) with siRNA-htt or scRNA. After 24 h, cells were transfected again with pARIS-mCherry-httQ23, pARIS-mCherry-httQ100, pARIS-mCherry-httQ23-Δdyn or pARIS-mCherry-httQ23-ΔHAP1. Cells were processed for western blotting or immunostaining 24 h after. DNA, siRNA and Lipofectamine 2000 quantities were used according to the manufacturer's instructions.

To perform co-immunoprecipitation experiments, HEK cells were transfected using Lipofectamine 2000 with siRNA-htt as described above. After 24 h the cells were transfected with pARIS-mCherry-Htt constructs and/or HAP1-GFP using the calcium phosphate method. Immunoprecipitation assays were performed after 24 h.

For videomicroscopy experiments mouse neuronal cells were electroporated with Kit L Nucleofector according to the supplier's manual (Amaxa, Köln, Germany). BDNF-eGFP and pARIS-htt DNA or equivalent amount of empty vector were added to the electroporation mix. After electroporation, cells were seeded in 12 well plates with 18 mm coverslips.

### Nocodazole treatment

Transfected HeLa GFP-mannosidase II cells were treated with 4 μM nocodazole for 30 min at 4°C and 90 min at 37°C to allow a complete depolymerization of microtubules. Cells were washed twice with DMEM prior to methanol fixation (2 min at -20°C).

### Antibodies

Anti-huntingtin antibodies used in this study htt-4C8, htt-2C1 and 1C2 were previously described [29,31], α-tubulin was from Sigma (St Louis, USA), high affinity anti-HA and anti-GFP were from Roche, anti-dynein intermediate chain (DIC) was from Chemicon (Billerica, USA), secondary IgG-HRP antibodies were from Jackson ImmunoResearch (WestGrove, USA), the mouse monoclonal antibody against the cis/medial Golgi marker CTR433 was previously described [42]. Alexa Fluor secondary antibodies used in immunofluorescence experiments were from Invitrogen.

### Western Blot

Transfected cells were harvested and lyzed in 50 mM Tris-HCl, pH 7.5, containing 0.1% Triton X-100, 2 mM EDTA, 2 mM EGTA, 50 mM NaF, 10 mM β-glycerophosphate, 5 mM sodium pyrophosphate, 1 mM sodium orthovanadate, 0.1% (v/v) β-mercaptoethanol, 250 μM PMSF, 10 mg/ml aprotinin and leupeptin. Cell lysates were centrifuged at 20,000 g for 10 min at 4°C. Equal amounts of protein were subjected to SDS-PAGE on 6% polyacrylamide gels and transferred to nitrocellulose membranes (Whatman, Dassel, Germany). Blocked membranes (5% milk in TBS-0.1% Tween-20) were incubated with mouse anti-huntingtin antibodies (htt-4C8, htt-2C1), mouse anti-polyQ expansion (1C2), rat anti-HA, mouse anti-GFP, mouse anti-DIC or mouse anti-α-tubulin antibodies and washed three times with TBS-0.1% Tween-20 for 10 min. Membranes were then labelled with secondary IgG-HRP antibodies raised against each corresponding primary antibody. After three washes, the membranes were incubated with SuperSignal West Pico Chemiluminescent Substrate (Pierce, Erembodegem, Belgium) according to the instructions of the supplier. Membranes were exposed to Amersham Hyperfilm™ MP (GE Healthcare, Buckinghamshire, UK) films and developed.

### Immunoprecipitation

Immunoprecipitations were performed as described [59] with minor modifications. Cell lysis and wash of the immunocomplexes were done in 50 mM of Tris 1 M (pH 8), 150 mM NaCl and 1% of NP40 containing protease and phosphatase inhibitors. Briefly, transfected cells were harvested and lyzed on ice. Lysates were centrifuged at 16,000 g (15 min at 4°C) and precleared (30 min at 4°C) using protein A-Sepharose beads (Sigma). Cleared lysates were incubated for 2-3 h at 4°C with protein A-Sepharose beads conjugated to mouse htt-4C8, mouse DIC or rat HA antibodies. Immunoprecipitates were washed three times and analyzed by Western blot as described.

### Immunofluorescence

After methanol fixation cells were blocked for 1 h at RT with PBS-BSA 3% and incubated with primary antibodies for 1 h prior staining with Alexa Fluor secondary antibodies. Nuclei were stained with DAPI (Roche). The mounting medium was 0.1 g/ml Mowiol 4-88 (Calbiochem, Darmstadt, Germany) in 20% glycerol.

### Image acquisition

Images on fixed samples were acquired at RT with a Leica SP5 laser scanning confocal microscope equipped with a 63 × oil-immersion objective or with a Leica DM RXA microscope with a PL APO oil 63 × NA of 1.4 objective coupled to a piezzo and a Micromax RTE/CCD-1300-Y/HS camera controlled by Metamorph software (Molecular Devices, Sunnyvale, CA). Z-stack step was of 0.2 μm. All stacks were treated by automatic batch deconvolution using the PSF of the optical system, Meinel algorithm with parameters set at 7 iterations, 0.7 sigma and 4 frequencies.

### Computer morphometric analysis of the Golgi apparatus

Images of fixed cells were acquired as described (see above, image aquisition). Only HeLa cells stably expressing GFP-mannosidase II and transfected with our pARIS-htt constructs were analyzed. Once deconvolved, images were analyzed with ImageJ software using 3D object counter plugin ([60]; available at http://imagejdocu.tudor.lu/doku.php?id=plugin:analysis:3d_object_counter:start). The quantification was achieved tagging each identified object within the z-stacks (around 30 z-stacks per image), treating each Golgi particle as an individual object. Statistics about each object were calculated, volume as: number of voxels of the object *× *x calibration *× *y calibration *× *z calibration. The overall measurements were obtained from 3 independent experiments and analyzed to determine the mean volume per particle for each condition.

### Videomicroscopy

Mouse neuronal transfected cells were grown on glass coverslips and mounted in a Ludin's chamber. The microscope and the chamber were kept at 33°C. Live videomicroscopy was carried out using a Leica DM IRBE microscope and a PL APO oil 100 × objective with a numerical aperture of 1.40-0.70, coupled to a piezo device (PI) and recorded with Photometrics CoolSNAP HQ2 camera (Roper Scientific, Trenton, NJ) controlled by Metamorph software. Images were collected in stream set at 2 × 2 binning with an exposure time of 50-150 ms (frequency of 2 s) with a Z-step of 0.3 μm. Deconvolution was performed as described for fixed samples. All dynamic parameters of intracellular transport were obtained from three independent experiments with a total of about 1500-5000 measures from 18-39 independent cells. Dynamics were characterized by tracking positions of eGFP vesicles as a function of time with an especially developed plugin (available at http://rsb.info.nih.gov/ij/plugins/track/track.html) for Image J.

## Abbreviations

The abbreviations used are BDNF: brain-derived neurotrophic factor; GA: Golgi apparatus; HD: Huntington's disease; htt: huntingtin; IP: immunoprecipitation; MT: microtubule; NZ: nocodazole; polyQ: polyglutamine; RT: room temperature; WB: western blot.

## Competing interests

The authors declare that they have no competing interests.

## Authors' contributions

RP, MMC, GK, SH and FS designed the experiments. RP, MMC and GP performed the experiments. RP, MMC, SH and FS analyzed the data. RP, MMC, SH and FS wrote the paper. All authors read and approved the final manuscript.

## Supplementary Material

Additional file 1 **Sequences and maps of pARIS-htt constructs used in the study**. The file includes vector maps, DNA sequences, protein translation and additional information for pARIS-mCherry-httQ23/Q100 plasmids in Entry vector. They were generated using Gene Construction Kit (Textco BioSoftware, West Lebanon, USA) and Serial Cloner 2.0 (available at http://serialbasics.free.fr/Serial_Cloner.html.) software. Click here for file

Additional file 2 **Sequence text files of pARIS-htt constructs used in the study**. It includes sequences of pARIS-mCherry-httQ23/Q100 plasmids in Entry and pcDNA vectors. Click here for file

## References

[B1] Borrell-Pages M Zala D Humbert S Saudou F Huntington's disease: from huntingtin function and dysfunction to therapeutic strategies Cell Mol Life Sci 2006 63 22 2642 2660 10.1007/s00018-006-6242-0 17041811 PMC11136202

[B2] Li S Li XJ Multiple pathways contribute to the pathogenesis of Huntington disease Mol Neurodegener 2006 1 1 19 10.1186/1750-1326-1-19 17173700 PMC1764744

[B3] Cattaneo E Zuccato C Tartari M Normal huntingtin function: an alternative approach to Huntington's disease Nat Rev Neurosci 2005 6 12 919 930 10.1038/nrn1806 16288298

[B4] Zuccato C Ciammola A Rigamonti D Leavitt BR Goffredo D Conti L MacDonald ME Friedlander RM Silani V Hayden MR Timmusk T Sipione S Cattaneo E Loss of huntingtin-mediated BDNF gene transcription in Huntington's disease Science 2001 293 5529 493 498 10.1126/science.1059581 11408619

[B5] Gauthier LR Charrin BC Borrell-Pages M Dompierre JP Rangone H Cordelieres FP De Mey J MacDonald ME Lessmann V Humbert S Saudou F Huntingtin controls neurotrophic support and survival of neurons by enhancing BDNF vesicular transport along microtubules Cell 2004 118 1 127 138 10.1016/j.cell.2004.06.018 15242649

[B6] Humbert S Bryson EA Cordelieres FP Connors NC Datta SR Finkbeiner S Greenberg ME Saudou F The IGF-1/Akt pathway is neuroprotective in Huntington's disease and involves Huntingtin phosphorylation by Akt Dev Cell 2002 2 6 831 837 10.1016/S1534-5807(02)00188-0 12062094

[B7] Rangone H Poizat G Troncoso J Ross CA MacDonald ME Saudou F Humbert S The serum- and glucocorticoid-induced kinase SGK inhibits mutant huntingtin-induced toxicity by phosphorylating serine 421 of huntingtin Eur J Neurosci 2004 19 2 273 279 10.1111/j.0953-816X.2003.03131.x 14725621

[B8] Pardo R Colin E Regulier E Aebischer P Deglon N Humbert S Saudou F Inhibition of calcineurin by FK506 protects against polyglutamine-huntingtin toxicity through an increase of huntingtin phosphorylation at S421 J Neurosci 2006 26 5 1635 1645 10.1523/JNEUROSCI.3706-05.2006 16452687 PMC6675484

[B9] Pineda JR Pardo R Zala D Yu H Humbert S Saudou F Genetic and pharmacological inhibition of calcineurin corrects the BDNF transport defect in Huntington's disease Mol Brain 2009 2 1 33 10.1186/1756-6606-2-33 19860865 PMC2776580

[B10] Colin E Regulier E Perrin V Durr A Brice A Aebischer P Deglon N Humbert S Saudou F Akt is altered in an animal model of Huntington's disease and in patients Eur J Neurosci 2005 21 6 1478 1488 10.1111/j.1460-9568.2005.03985.x 15845076

[B11] Warby SC Chan EY Metzler M Gan L Singaraja RR Crocker SF Robertson HA Hayden MR Huntingtin phosphorylation on serine 421 is significantly reduced in the striatum and by polyglutamine expansion in vivo Hum Mol Genet 2005 14 11 1569 1577 10.1093/hmg/ddi165 15843398

[B12] Colin E Zala D Liot G Rangone H Borrell-Pages M Li XJ Saudou F Humbert S Huntingtin phosphorylation acts as a molecular switch for anterograde/retrograde transport in neurons Embo J 2008 27 15 2124 2134 10.1038/emboj.2008.133 18615096 PMC2516882

[B13] Zala D Colin E Rangone H Liot G Humbert S Saudou F Phosphorylation of mutant huntingtin at S421 restores anterograde and retrograde transport in neurons Hum Mol Genet 2008 15; 17 24 3837 46 10.1093/hmg/ddn281 18772195

[B14] Graham RK Deng Y Slow EJ Haigh B Bissada N Lu G Pearson J Shehadeh J Bertram L Murphy Z Warby SC Doty CN Roy S Wellington CL Leavitt BR Raymond LA Nicholson DW Hayden MR Cleavage at the caspase-6 site is required for neuronal dysfunction and degeneration due to mutant huntingtin Cell 2006 125 6 1179 1191 10.1016/j.cell.2006.04.026 16777606

[B15] Luo S Vacher C Davies JE Rubinsztein DC Cdk5 phosphorylation of huntingtin reduces its cleavage by caspases: implications for mutant huntingtin toxicity J Cell Biol 2005 169 4 647 656 10.1083/jcb.200412071 15911879 PMC2171695

[B16] Schilling B Gafni J Torcassi C Cong X Row RH Lafevre-Bernt MA Cusack MP Ratovitski T Hirschhorn R Ross CA Gibson BW Ellerby LM Huntingtin phosphorylation sites mapped by mass spectrometry: Modulation of cleavage and toxicity J Biol Chem 2006 281 23686 23697 10.1074/jbc.M513507200 16782707

[B17] Jeong H Then F Melia TJ Jr Mazzulli JR Cui L Savas JN Voisine C Paganetti P Tanese N Hart AC Yamamoto A Krainc D Acetylation targets mutant huntingtin to autophagosomes for degradation Cell 2009 137 1 60 72 10.1016/j.cell.2009.03.018 19345187 PMC2940108

[B18] Yanai A Huang K Kang R Singaraja RR Arstikaitis P Gan L Orban PC Mullard A Cowan CM Raymond LA Drisdel RC Green WN Ravikumar B Rubinsztein DC El-Husseini A Hayden MR Palmitoylation of huntingtin by HIP14 is essential for its trafficking and function Nat Neurosci 2006 9 6 824 831 10.1038/nn1702 16699508 PMC2279235

[B19] Andrade MA Bork P HEAT repeats in the Huntington's disease protein Nat Genet 1995 11 2 115 116 10.1038/ng1095-115 7550332

[B20] Takano H Gusella JF The predominantly HEAT-like motif structure of huntingtin and its association and coincident nuclear entry with dorsal, an NF-kB/Rel/dorsal family transcription factor BMC Neurosci 2002 3 1 15 10.1186/1471-2202-3-15 12379151 PMC137586

[B21] Palidwor GA Shcherbinin S Huska MR Rasko T Stelzl U Arumughan A Foulle R Porras P Sanchez-Pulido L Wanker EE Andrade-Navarro MA Detection of alpha-rod protein repeats using a neural network and application to huntingtin PLoS Comput Biol 2009 5 3 e1000304 10.1371/journal.pcbi.1000304 19282972 PMC2647740

[B22] Grinthal A Adamovic I Weiner B Karplus M Kleckner N PR65, the HEAT-repeat scaffold of phosphatase PP2A, is an elastic connector that links force and catalysis Proc Natl Acad Sci USA 2010 107 6 2467 2472 10.1073/pnas.0914073107 20133745 PMC2823866

[B23] Goehler H Lalowski M Stelzl U Waelter S Stroedicke M Worm U Droege A Lindenberg KS Knoblich M Haenig C Herbst M Suopanki J Scherzinger E Abraham C Bauer B Hasenbank R Fritzsche A Ludewig AH Bussow K Coleman SH Gutekunst CA Landwehrmeyer BG Lehrach H Wanker EE A protein interaction network links GIT1, an enhancer of huntingtin aggregation, to Huntington's disease Mol Cell 2004 15 6 853 865 10.1016/j.molcel.2004.09.016 15383276

[B24] Kaltenbach LS Romero E Becklin RR Chettier R Bell R Phansalkar A Strand A Torcassi C Savage J Hurlburt A Cha GH Ukani L Chepanoske CL Zhen Y Sahasrabudhe S Olson J Kurschner C Ellerby LM Peltier JM Botas J Hughes RE Huntingtin interacting proteins are genetic modifiers of neurodegeneration PLoS Genet 2007 3 5 e82 10.1371/journal.pgen.0030082 17500595 PMC1866352

[B25] Mangiarini L Sathasivam K Seller M Cozens B Harper A Hetherington C Lawton M Trottier Y Lehrach H Davies SW Bates GP Exon 1 of the HD gene with an expanded CAG repeat is sufficient to cause a progressive neurological phenotype in transgenic mice Cell 1996 87 3 493 506 10.1016/S0092-8674(00)81369-0 8898202

[B26] Peters MF Ross CA Preparation of human cDNas encoding expanded polyglutamine repeats Neurosci Lett 1999 275 2 129 132 10.1016/S0304-3940(99)00758-2 10568516

[B27] Gaietta G Deerinck TJ Adams SR Bouwer J Tour O Laird DW Sosinsky GE Tsien RY Ellisman MH Multicolor and electron microscopic imaging of connexin trafficking Science 2002 296 5567 503 507 10.1126/science.1068793 11964472

[B28] Ju W Morishita W Tsui J Gaietta G Deerinck TJ Adams SR Garner CC Tsien RY Ellisman MH Malenka RC Activity-dependent regulation of dendritic synthesis and trafficking of AMPA receptors Nat Neurosci 2004 7 3 244 253 10.1038/nn1189 14770185

[B29] Trottier Y Devys D Imbert G Saudou F An I Lutz Y Weber C Agid Y Hirsch EC Mandel JL Cellular localization of the Huntington's disease protein and discrimination of the normal and mutated form Nat Genet 1995 10 1 104 110 10.1038/ng0595-104 7647777

[B30] Cong SY Pepers BA Roos RA Van Ommen GJ Dorsman JC Epitope mapping of monoclonal antibody 4C8 recognizing the protein huntingtin Hybridoma (Larchmt) 2005 24 5 231 235 10.1089/hyb.2005.24.231 16225422

[B31] Trottier Y Lutz Y Stevanin G Imbert G Devys D Cancel G Saudou F Weber C David G Tora L Polyglutamine expansion as a pathological epitope in Huntington's disease and four dominant cerebellar ataxias Nature 1995 378 6555 403 406 10.1038/378403a0 7477379

[B32] del Toro D Canals JM Gines S Kojima M Egea G Alberch J Mutant huntingtin impairs the post-Golgi trafficking of brain-derived neurotrophic factor but not its Val66Met polymorphism J Neurosci 2006 26 49 12748 12757 10.1523/JNEUROSCI.3873-06.2006 17151278 PMC6674849

[B33] Strehlow AN Li JZ Myers RM Wild-type huntingtin participates in protein trafficking between the Golgi and the extracellular space Hum Mol Genet 2007 16 4 391 409 10.1093/hmg/ddl467 17189290

[B34] Caviston JP Ross JL Antony SM Tokito M Holzbaur EL Huntingtin facilitates dynein/dynactin-mediated vesicle transport Proc Natl Acad Sci USA 2007 104 24 10045 10050 10.1073/pnas.0610628104 17548833 PMC1891230

[B35] Corthesy-Theulaz I Pauloin A Pfeffer SR Cytoplasmic dynein participates in the centrosomal localization of the Golgi complex J Cell Biol 1992 118 6 1333 1345 10.1083/jcb.118.6.1333 1387874 PMC2289611

[B36] Echeverri CJ Paschal BM Vaughan KT Vallee RB Molecular characterization of the 50-kD subunit of dynactin reveals function for the complex in chromosome alignment and spindle organization during mitosis J Cell Biol 1996 132 4 617 633 10.1083/jcb.132.4.617 8647893 PMC2199864

[B37] Thyberg J Moskalewski S Role of microtubules in the organization of the Golgi complex Exp Cell Res 1999 246 2 263 279 10.1006/excr.1998.4326 9925741

[B38] Burkhardt JK Echeverri CJ Nilsson T Vallee RB Overexpression of the dynamitin (p50) subunit of the dynactin complex disrupts dynein-dependent maintenance of membrane organelle distribution J Cell Biol 1997 139 2 469 484 10.1083/jcb.139.2.469 9334349 PMC2139801

[B39] He Y Francis F Myers KA Yu W Black MM Baas PW Role of cytoplasmic dynein in the axonal transport of microtubules and neurofilaments J Cell Biol 2005 168 5 697 703 10.1083/jcb.200407191 15728192 PMC2171826

[B40] Levy JR Sumner CJ Caviston JP Tokito MK Ranganathan S Ligon LA Wallace KE LaMonte BH Harmison GG Puls I Fischbeck KH Holzbaur EL A motor neuron disease-associated mutation in p150Glued perturbs dynactin function and induces protein aggregation J Cell Biol 2006 172 5 733 745 10.1083/jcb.200511068 16505168 PMC2063705

[B41] Gomez M Scales SJ Kreis TE Perez F Membrane recruitment of coatomer and binding to dilysine signals are separate events J Biol Chem 2000 275 37 29162 29169 10.1074/jbc.M003630200 10864930

[B42] Jasmin BJ Cartaud J Bornens M Changeux JP Golgi apparatus in chick skeletal muscle: changes in its distribution during end plate development and after denervation Proc Natl Acad Sci USA 1989 86 18 7218 7222 10.1073/pnas.86.18.7218 2674951 PMC298028

[B43] Cole NB Sciaky N Marotta A Song J Lippincott-Schwartz J Golgi dispersal during microtubule disruption: regeneration of Golgi stacks at peripheral endoplasmic reticulum exit sites Mol Biol Cell 1996 7 4 631 650 8730104 10.1091/mbc.7.4.631 PMC275914

[B44] Li XJ Li SH Sharp AH Nucifora FC Jr Schilling G Lanahan A Worley P Snyder SH Ross CA A huntingtin-associated protein enriched in brain with implications for pathology Nature 1995 378 6555 398 402 10.1038/378398a0 7477378

[B45] Engelender S Sharp AH Colomer V Tokito MK Lanahan A Worley P Holzbaur EL Ross CA Huntingtin-associated protein 1 (HAP1) interacts with the p150Glued subunit of dynactin Hum Mol Genet 1997 6 13 2205 2212 10.1093/hmg/6.13.2205 9361024

[B46] Li SH Gutekunst CA Hersch SM Li XJ Interaction of huntingtin-associated protein with dynactin P150Glued J Neurosci 1998 18 4 1261 1269 9454836 10.1523/JNEUROSCI.18-04-01261.1998 PMC6792727

[B47] Bertaux F Sharp AH Ross CA Lehrach H Bates GP Wanker E HAP1-huntingtin interactions do not contribute to the molecular pathology in Huntington's disease transgenic mice FEBS Lett 1998 426 2 229 232 10.1016/S0014-5793(98)00352-4 9599014

[B48] McGuire JR Rong J Li SH Li XJ Interaction of Huntingtin-associated protein-1 with kinesin light chain: implications in intracellular trafficking in neurons J Biol Chem 2006 281 6 3552 3559 10.1074/jbc.M509806200 16339760

[B49] Dompierre JP Godin JD Charrin BC Cordelieres FP King SJ Humbert S Saudou F Histone deacetylase 6 inhibition compensates for the transport deficit in Huntington's disease by increasing tubulin acetylation J Neurosci 2007 27 13 3571 3583 10.1523/JNEUROSCI.0037-07.2007 17392473 PMC6672116

[B50] Caviston JP Holzbaur EL Huntingtin as an essential integrator of intracellular vesicular trafficking Trends Cell Biol 2009 19 4 147 155 10.1016/j.tcb.2009.01.005 19269181 PMC2930405

[B51] Wanker EE Rovira C Scherzinger E Hasenbank R Walter S Tait D Colicelli J Lehrach H HIP-I: a huntingtin interacting protein isolated by the yeast two-hybrid system Hum Mol Genet 1997 6 3 487 495 10.1093/hmg/6.3.487 9147654

[B52] Pal A Severin F Lommer B Shevchenko A Zerial M Huntingtin-HAP40 complex is a novel Rab5 effector that regulates early endosome motility and is up-regulated in Huntington's disease J Cell Biol 2006 172 4 605 618 10.1083/jcb.200509091 16476778 PMC2063679

[B53] Hattula K Peranen J FIP-2, a coiled-coil protein, links huntingtin to Rab8 and modulates cellular morphogenesis Curr Biol 2000 24 1603 1606 10.1016/S0960-9822(00)00864-2 11137014

[B54] Li X Standley C Sapp E Valencia A Qin ZH Kegel KB Yoder J Comer-Tierney LA Esteves M Chase K Alexander J Masso N Sobin L Bellve K Tuft R Lifshitz L Fogarty K Aronin N DiFiglia M Mutant huntingtin impairs vesicle formation from recycling endosomes by interfering with Rab11 activity Mol Cell Biol 2009 29 22 6106 6116 10.1128/MCB.00420-09 19752198 PMC2772576

[B55] Faber PW Barnes GT Srinidhi J Chen J Gusella JF MacDonald ME Huntingtin interacts with a family of WW domain proteins Hum Mol Genet 1998 7 9 1463 1474 10.1093/hmg/7.9.1463 9700202

[B56] Haubensak W Narz F Heumann R Lessmann V BDNF-GFP containing secretory granules are localized in the vicinity of synaptic junctions of cultured cortical neurons J Cell Sci 1998 111 1483 1493 9580557 10.1242/jcs.111.11.1483

[B57] Trettel F Rigamonti D Hilditch-Maguire P Wheeler VC Sharp AH Persichetti F Cattaneo E MacDonald ME Dominant phenotypes produced by the HD mutation in STHdh(Q111) striatal cells Hum Mol Genet 2000 9 19 2799 2809 10.1093/hmg/9.19.2799 11092756

[B58] Saudou F Finkbeiner S Devys D Greenberg ME Huntingtin acts in the nucleus to induce apoptosis but death does not correlate with the formation of intranuclear inclusions Cell 1998 95 55 66 10.1016/S0092-8674(00)81782-1 9778247

[B59] Crespo PM Silvestre DC Gil GA Maccioni HJ Daniotti JL Caputto BL c-Fos activates glucosylceramide synthase and glycolipid synthesis in PC12 cells J Biol Chem 2008 283 45 31163 31171 10.1074/jbc.M709257200 18784083 PMC2662181

[B60] Bolte S Cordelieres FP A guided tour into subcellular colocalization analysis in light microscopy J Microsc 2006 224 Pt 3 213 232 10.1111/j.1365-2818.2006.01706.x 17210054

