# Supplementary material for: pARIS-htt: an optimised expression platform to study huntingtin reveals functional domains required for vesicular trafficking
Source: Mol Brain. 2010 Jun 1;3:17. doi: 10.1186/1756-6606-3-17 (PMC2887845; doi:10.1186/1756-6606-3-17)
Supplement: Additional file 1 — Sequences and maps of pARIS-htt constructs used in the study. The file includes vector maps, DNA sequences, protein translation and additional information for pARIS-mCherry-httQ23/Q100 plasmids in Entry vector. They were generated using Gene Construction Kit (Textco BioSoftware, West Lebanon, USA) and Serial Cloner 2.0 (available at http://serialbasics.free.fr/Serial_Cloner.html.) software. [file 1756-6606-3-17-S1.PDF]

Full name : pARIS-htt-N[His-Cherry]Q23-C[HA-TC]
Used name : pARIS-mCherry-httQ23
vector : pENTRY

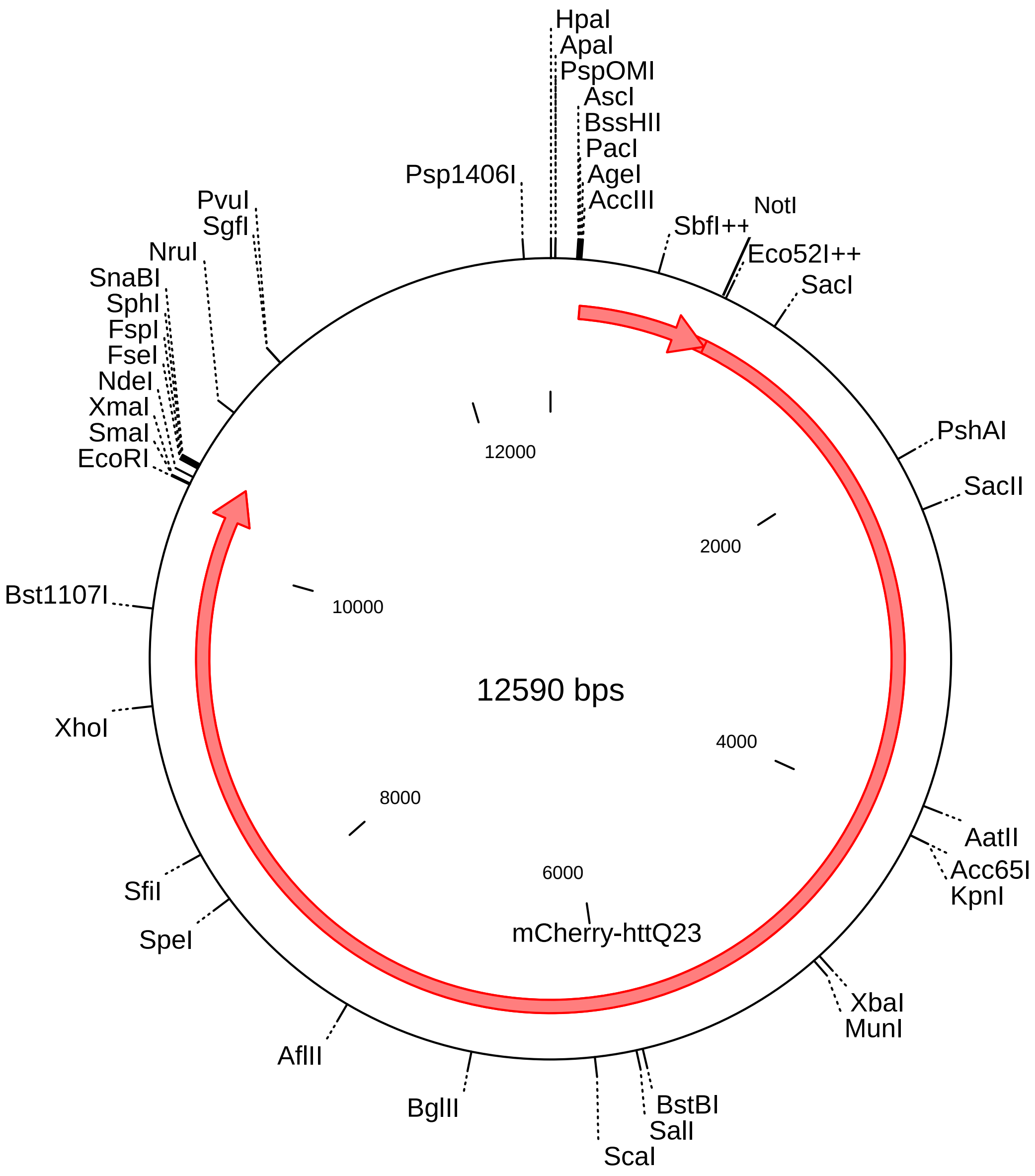

gene : mCherry-httQ23
- start : 165
- end 10451

Molecule: pARIS-mCherry-httQ23, 12590 bps DNA Circular
Description:
File Name: pARIS-mCherry-httQ23.cm5, dated 23 Apr 2010
Printed: List of Sites in bps, sorted by Enzyme name.
Filter ON: cut N <= 1

	#sites	----- Bp position of recognition site -----
AatII	1	3900
Acc65I	1	4061
AccIII	1	155
AflII	1	7361
AgeI	1	149
ApaI	1	24
AscI	1	133
BglII	1	6692
BssHII	1	134
Bst1107I	1	9695
BstBI	1	5829
Ecl136II	1	1188
Eco52I	1	907
EcoRI	1	10344
FseI	1	10434
FspI	1	10443
HpaI	1	3
KpnI	1	4061
MunI	1	4857
NdeI	1	10386
NotI	1	906
NruI	1	10767
PacI	1	141
PshAI	1	2102
Psp1406I	1	12457
PspOMI	1	24
PvuI	1	11109
SacI	1	1188
SacII	1	2383
SalI	1	5862
SbfI	1	548
ScaI	1	6072
SfiI	1	8420
SgfI	1	11108
SgrAI	1	878
SmaI	1	10350
SnaBI	1	10458
SpeI	1	8156
SphI	1	10452
XbaI	1	4821
XhoI	1	9206
XmaI	1	10350

attL1

```
1    gcgttaacgctagcatggatctcgggccccaaataatgattttattttgactgatagtgacctgttcgttgcaa
                                                                      AscI       PacI
75   caaattgatgagcaatgcttttttataatgccaactttGTACAAAAAAGCAGGCTGAAGGCGCGCCTTAATTAA
     AgeI    BspEI                 histidine tag            alanine hinge   mCherry →
149  ACCGGTTCCGGACACCATGGCCCACCACCATCACCACCATGCCGCCGCTGCCGTGAGCAAGGGCGAGGAGG
                   1▶ M  A  H  H  H  H  H  H  A  A  A  A  V  S  K  G  E  E
220  ATAACATGGCCATCATCAAGGAGTTCATGCGCTTCAAGGTGCACATGGAGGGCTCCGTGAACGGCCACGAGTTC
19▶  D  N  M  A  I  I  K  E  F  M  R  F  K  V  H  M  E  G  S  V  N  G  H  E  F
294  GAGATCGAGGGCGAGGGCGAGGGCCGCCCCTACGAGGGCACCCAGACCGCCAAGCTGAAGGTGACCAAGGGTGG
44▶   E  I  E  G  E  G  E  G  R  P  Y  E  G  T  Q  T  A  K  L  K  V  T  K  G  G
368  CCCCCTGCCCTTCGCCTGGGACATCCTGTCCCCTCAGTTCATGTACGGCTCCAAGGCCTACGTGAAGCACCCCG
68▶   P  L  P  F  A  W  D  I  L  S  P  Q  F  M  Y  G  S  K  A  Y  V  K  H  P
442  CCGACATCCCCGACTACTTGAAGCTGTCCTTCCCCGAGGGCTTCAAGTGGGAGCGCGTGATGAACTTCGAGGAC
93▶  A  D  I  P  D  Y  L  K  L  S  F  P  E  G  F  K  W  E  R  V  M  N  F  E  D     His Cherry
516  GGCGGCGTGGTGACCGTGACCCAGGACTCCTCCCTGCAGGACGGCGAGTTCATCTACAAGGTGAAGCTGCGCGG
118▶  G  G  V  V  T  V  T  Q  D  S  S  L  Q  D  G  E  F  I  Y  K  V  K  L  R  G
590  CACCAACTTCCCCTCCGACGGCCCCGTAATGCAGAAGAAGACCATGGGCTGGGAGGCCTCCTCCGAGCGGATGT
142▶  T  N  F  P  S  D  G  P  V  M  Q  K  K  T  M  G  W  E  A  S  S  E  R  M
664  ACCCCGAGGACGGCGCCCTGAAGGGCGAGATCAAGCAGAGGCTGAAGCTGAAGGACGGCGGCCACTACGACGCT
167▶ Y  P  E  D  G  A  L  K  G  E  I  K  Q  R  L  K  L  K  D  G  G  H  Y  D  A
738  GAGGTCAAGACCACCTACAAGGCCAAGAAGCCCGTGCAGCTGCCCGGCGCCTACAACGTCAACATCAAGTTGGA
192▶  E  V  K  T  T  Y  K  A  K  K  P  V  Q  L  P  G  A  Y  N  V  N  I  K  L  D
812  CATCACCTCCCACAACGAGGACTACACCATCGTGGAACAGTACGAACGCGCCGAGGGCCGCCACTCCACCGGCG
216▶  I  T  S  H  N  E  D  Y  T  I  V  F  Q  Y  F  R  A  E  G  R  H  S  T  G
                         NotI ala hinge    human huntingtin →
886  GCATGGACGAGCTGTACAAGGCGGCCGCCGCGACCCTGGAAAAGCTGATGAAGGCCTTCGAGTCCCTC
241▶ G  M  D  E  L  Y  K  A  A  A  A  T  L  E  K  L  M  K  A  F  E  S  L     poly Q
954  AAGTCCTTCCAGCAACAGCAACAGCAACAGCAACAGCAACAGCAACAGCAACAGCAGCAACAGCAGCAA     Stretch
264▶  K  S  F  Q  Q  Q  Q  Q  Q  Q  Q  Q  Q  Q  Q  Q  Q  Q  Q  Q  Q          23Q /
1017 CAGCAGCAACAACAGCCGCCACCGCCACCGCCGCCACCGCCGCCACCTCAGCTTCCTCAGCCG           100 Q
285▶  Q  Q  Q  Q  Q  P  P  P  P  P  P  P  P  P  P  P  Q  L  P  Q  P
1080 CCGCCGCAGGCACAGCCGCTGCTGCCTCAGCCGCAGCCGCCCCCTCCGCCGCCCCCGCCACCA
306▶  P  P  Q  A  Q  P  L  L  P  Q  P  Q  P  P  P  P  P  P  P  P  P
                                                      SacI
1143 CCCGGCCCGGCTGTGGCTGAGGAGCCGCTGCACCGACCAAAGAAGGAGCTCAGTGCAACTAAG     SiHtt-
327▶  P  G  P  A  V  A  E  E  P  L  H  R  P  K  K  E  L  S  A  T  K      hu585 /
1206 AAAGACCGTGTGAATCATTGTCTGACAATATGTGAAAACATAGTGGCACAGTCTGTCAGAAAT     siHtt-1.1
348▶  K  D  R  V  N  H  C  L  T  I  C  E  N  I  V  A  Q  S  V  R  N
1269 TCTCCAGAATTTCAGAAACTTCTGGGCATCGCTATGGAACTTTTTCTGCTGTGCAGTGATGAC
369▶  S  P  E  F  Q  K  L  L  G  I  A  M  E  L  F  L  L  C  S  D  D
1332 GCAGAGTCAGATGTCAGGATGGTGGCTGACGAATGCCTCAACAAAGTTATCAAGGCACTCATG     SiHtt-6
390▶  A  E  S  D  V  R  M  V  A  D  E  C  L  N  K  V  I  K  A  L  M
1395 GACAGCAACCTTCCAAGGTTACAGCTCGAACTGTATAAGGAAATTAAAAAGAATGGTGCCCCT
411▶  D  S  N  L  P  R  L  Q  L  E  L  Y  K  E  I  K  K  N  G  A  P
1458 CGGAGTTTGCGTGCTGCCCTGTGGAGGTTTGCTGAGCTGGCTCACCTGGTTCGGCCTCAGAAA
432▶  R  S  L  R  A  A  L  W  R  F  A  E  L  A  H  L  V  R  P  Q  K
1521 TGCAGGCCTTACCTGGTGAACCTTCTGCCGTGCCTGACTCGAACAAGCAAGAGACCCGAAGAA
453▶  C  R  P  Y  L  V  N  L  L  P  C  L  T  R  T  S  K  R  P  E  E        Htt
1584 TCAGTCCAGGAGACCTTGGCTGCAGCTGTTCCCAAAATTATGGCTTCTTTTGGCAATTTTGCA
474▶  S  V  Q  E  T  L  A  A  A  V  P  K  I  M  A  S  F  G  N  F  A
1647 AATGACAATGAAATTAAGGTTTTGTTAAAGGCCTTCATAGCGAACCTGAAGTCAAGCTCCCCC
495▶  N  D  N  E  I  K  V  L  L  K  A  F  I  A  N  L  K  S  S  S  P
1710 ACCATTCGGCGGACAGCGGCTGGATCAGCAGTGAGCATCTGCCAGCACTCAAGAAGGACACAA
516▶  T  I  R  R  T  A  A  G  S  A  V  S  I  C  Q  H  S  R  R  T  Q
1773 TATTTCTATAGTTGGCTACTAAATGTGCTCTTAGGCTTACTCGTTCCTGTCGAGGATGAACAC
537▶  Y  F  Y  S  W  L  L  N  V  L  L  G  L  L  V  P  V  E  D  E  H
1836 TCCACTCTGCTGATTCTTGGCGTGCTGCTCACCCTGAGGTATTTGGTGCCCTTGCTGCAGCAG
558▶  S  T  L  L  I  L  G  V  L  L  T  L  R  Y  L  V  P  L  L  Q  Q
1899 CAGGTCAAGGACACAAGCCTGAAAGGCAGCTTCGGAGTGACAAGGAAAGAAATGGAAGTCTCT
579▶  Q  V  K  D  T  S  L  K  G  S  F  G  V  T  R  K  E  M  E  V  S
1962 CCTTCTGCAGAGCAATTAGTGCAAGTCTACGAACTGACGTTACATCATACACAGCACCAAGAC     SiHtt-13
600▶  P  S  A  E  Q  L  V  Q  V  Y  E  L  T  L  H  H  T  Q  H  Q  D
```

```
2025 CACAATGTTGTGACCGGAGCCCTGGAGCTGTTGCAGCAGCTCTTCAGAACGCCTCCACCCGAG
 621▶ H  N  V  V  T  G  A  L  E  L  L  Q  Q  L  F  R  T  P  P  P  E
2088 CTTCTGCAAACCCTGACCGCAGTCGGGGGCATTGGGCAGCTCACCGCTGCTAAGGAGGAGTCT
 642▶ L  L  Q  T  L  T  A  V  G  G  I  G  Q  L  T  A  A  K  E  E  S
2151 GGTGGCCGAAGCCGTAGTGGGAGTATTGTGGAACTTATAGCTGGAGGGGGTTCCTCATGCAGC
 663▶ G  G  R  S  R  S  G  S  I  V  E  L  I  A  G  G  G  S  S  C  S
2214 CCTGTCCTTTCAAGAAAACAAAAAGGCAAAGTGCTCTTAGGAGAAGAAGAAGCCTTGGAGGAT
 684▶ P  V  L  S  R  K  Q  K  G  K  V  L  L  G  E  E  E  A  L  E  D
2277 GACTCTGAATCGAGATCGGATGTCAGCAGCTCTGCCTTAACAGCCTCAGTGAAGGATGAGATC
 705▶ D  S  E  S  R  S  D  V  S  S  S  A  L  T  A  S  V  K  D  E  I
                                                       SacII
2340 AGTGGAGAGCTGGCTGCTTCTTCAGGGGTTTCCACTCCAGGGTCCGCGGGGCATGACATCATC
 726▶ S  G  E  L  A  A  S  S  G  V  S  T  P  G  S  A  G  H  D  I  I
2403 ACAGAACAGCCACGGTCACAGCACACACTGCAGGCGGACTCAGTGGATCTGGCCAGCTGTGAC
 747▶ T  E  Q  P  R  S  Q  H  T  L  Q  A  D  S  V  D  L  A  S  C  D
2466 TTGACAAGCTCTGCCACTGATGGGGATGAGGAGGATATATTGAGCCACAGCTCCAGCCAGGTC
 768▶ L  T  S  S  A  T  D  G  D  E  E  D  I  L  S  H  S  S  S  Q  V
2529 AGCGCCGTCCCATCTGACCCTGCCATGGACCTGAATGATGGGACCCAGGCCTCGTCGCCCATC
 789▶ S  A  V  P  S  D  P  A  M  D  L  N  D  G  T  Q  A  S  S  P  I
2592 AGCGACAGCTCCCAGACCACCACCGAAGGGCCTGATTCAGCTGTTACCCCTTCAGACAGTTCT
 810▶ S  D  S  S  Q  T  T  T  E  G  P  D  S  A  V  T  P  S  D  S  S
2655 GAAATTGTGTTAGACGGCACCGACAACCAGTATTTGGGCCTGCAGATTGGACAGCCCCAGGAT
 831▶ E  I  V  L  D  G  T  D  N  Q  Y  L  G  L  Q  I  G  Q  P  Q  D
2718 GAAGATGAGGAAGCCACAGGTATTCTTCCTGATGAAGCCTCGGAGGCCTTCAGGAACTCTTCC
 852▶ E  D  E  E  A  T  G  I  L  P  D  E  A  S  E  A  F  R  N  S  S
2781 ATGGCCCTTCAACAGGCACATTTATTGAAAAACATGAGTCACTGCAGGCAGCCTTCTGACAGC
 873▶ M  A  L  Q  Q  A  H  L  L  K  N  M  S  H  C  R  Q  P  S  D  S
2844 AGTGTTGATAAATTTGTGTTGAGAGATGAAGCTACTGAACCGGGTGATCAAGAAAACAAGCCT
 894▶ S  V  D  K  F  V  L  R  D  E  A  T  E  P  G  D  Q  E  N  K  P
2907 TGCCGCATCAAAGGTGACATTGGACAGTCCACTGATGATGACTCTGCACCTCTTGTCCATTGT
 915▶ C  R  I  K  G  D  I  G  Q  S  T  D  D  D  S  A  P  L  V  H  C
2970 GTCCGCCTTTTATCTGCTTCGTTTTTGCTAACAGGGGGAAAAAATGTGCTGGTTCCCGACAGG
 936▶ V  R  L  L  S  A  S  F  L  L  T  G  G  K  N  V  L  V  P  D  R
3033 GATGTGAGGGTCAGCGTGAAGGCCCTGGCCCTCAGCTGTGTGGGAGCAGCTGTGGCCCTCCAC
 957▶ D  V  R  V  S  V  K  A  L  A  L  S  C  V  G  A  A  V  A  L  H
3096 CCGGAATCTTTCTTCAGCAAACTCTATAAAGTTCCTCTTGACACCACGGAATACCCTGAGGAA
 978▶ P  E  S  F  F  S  K  L  Y  K  V  P  L  D  T  T  E  Y  P  E  E
3159 CAGTATGTCTCAGACATCTTGAACTACATCGATCATGGAGACCCACAGGTTCGAGGAGCCACT
 999▶ Q  Y  V  S  D  I  L  N  Y  I  D  H  G  D  P  Q  V  R  G  A  T
3222 GCCATTCTCTGTGGGACCCTCATCTGCTCCATCCTCAGCAGGTCCCGCTTCCACGTGGGAGAT
1020▶ A  I  L  C  G  T  L  I  C  S  I  L  S  R  S  R  F  H  V  G  D
3285 TGGATGGGCACCATTAGAACCCTCACAGGAAATACATTTTCTTTGGCGGATTGCATTCCTTTG
1041▶ W  M  G  T  I  R  T  L  T  G  N  T  F  S  L  A  D  C  I  P  L
3348 CTGCGGAAAACACTGAAGGATGAGTCTTCTGTTACTTGCAAGTTAGCTTGCACAGCTGTGAGG
1062▶ L  R  K  T  L  K  D  E  S  S  V  T  C  K  L  A  C  T  A  V  R
3411 AACTGTGTCATGAGTCTCTGCAGCAGCAGCTACAGTGAGTTAGGACTGCAGCTGATCATCGAT
1083▶ N  C  V  M  S  L  C  S  S  S  Y  S  E  L  G  L  Q  L  I  I  D
3474 GTGCTGACTCTGAGGAACAGTTCCTATTGGCTGGTGAGGACAGAGCTTCTGGAAACCCTTGCA
1104▶ V  L  T  L  R  N  S  S  Y  W  L  V  R  T  E  L  L  E  T  L  A
3537 GAGATTGACTTCAGGCTGGTGAGCTTTTTGGAGGCAAAAGCAGAAAACTTACACAGAGGGGCT
1125▶ E  I  D  F  R  L  V  S  F  L  E  A  K  A  E  N  L  H  R  G  A
3600 CATCATTATACAGGGCTTTTAAAACTGCAAGAACGAGTGCTCAATAATGTTGTCATCCATTTG
1146▶ H  H  Y  T  G  L  L  K  L  Q  E  R  V  L  N  N  V  V  I  H  L
3663 CTTGGAGATGAAGACCCCAGGGTGCGACATGTTGCCGCAGCATCACTAATTAGGCTTGTCCCA
1167▶ L  G  D  E  D  P  R  V  R  H  V  A  A  A  S  L  I  R  L  V  P
3726 AAGCTGTTTTATAAATGTGACCAAGGACAAGCTGATCCAGTAGTGGCCGTGGCAAGAGATCAA
1188▶ K  L  F  Y  K  C  D  Q  G  Q  A  D  P  V  V  A  V  A  R  D  Q
3789 AGCAGTGTTTACCTGAAACTTCTCATGCATGAGACGCAGCCTCCATCTCATTTCTCCGTCAGC
1209▶ S  S  V  Y  L  K  L  L  M  H  E  T  Q  P  P  S  H  F  S  V  S
3852 ACAATAACCAGAATATATAGAGGCTATAACCTACTACCAAGCATAACAGACGTCACTATGGAA
1230▶ T  I  T  R  I  Y  R  G  Y  N  L  L  P  S  I  T  D  V  T  M  E
3915 AATAACCTTTCAAGAGTTATTGCAGCAGTTTCTCATGAACTAATCACATCAACCACCAGAGCA
1251▶ N  N  L  S  R  V  I  A  A  V  S  H  E  L  I  T  S  T  T  R  A
```

Htt

```
3978 CTCACATTTGGATGCTGTGAAGCCTTGTGTCTTCTTTCCACTGCCTTCCCAGTTTGCATTTGG
1272▸ L  T  F  G  C  C  E  A  L  C  L  L  S  T  A  F  P  V  C  I  W
                            KpnI
4041 AGTTTAGGTTGGCACTGTGGGGTACCGCCACTGAGTGCCTCAGATGAGTCTAGGAAGAGCTGT
1293▸ S  L  G  W  H  C  G  V  P  P  L  S  A  S  D  E  S  R  K  S  C
4104 ACCGTTGGGATGGCCACAATGATTCTGACCCTGCTCTCGTCAGCTTGGTTCCCATTGGATCTC
1314▸ T  V  G  M  A  T  M  I  L  T  L  L  S  S  A  W  F  P  L  D  L
4167 TCAGCCCATCAAGATGCTTTGATTTTGGCCGGAAACTTGCTTGCAGCCAGTGCTCCCAAATCT
1335▸ S  A  H  Q  D  A  L  I  L  A  G  N  L  L  A  A  S  A  P  K  S
4230 CTGAGAAGTTCATGGGCCTCTGAAGAAGAAGCCAACCCAGCAGCCACCAAGCAAGAGGAGGTC
1356▸ L  R  S  S  W  A  S  E  E  E  A  N  P  A  A  T  K  Q  E  E  V
4293 TGGCCAGCCCTGGGGGACAGAGCCCTGGTGCCCATGGTGGAGCAGCTCTTCTCTCACCTGCTG
1377▸ W  P  A  L  G  D  R  A  L  V  P  M  V  E  Q  L  F  S  H  L  L
4356 AAGGTGATTAACATTTGTGCCCACGTCCTGGATGACGTGGCTCCTGGACCCGCAATAAAGGCA
1398▸ K  V  I  N  I  C  A  H  V  L  D  D  V  A  P  G  P  A  I  K  A
4419 GCCTTGCCTTCTCTAACAAACCCCCCTTCTCTAAGTCCCATCCGACGAAAGGGGAAGGAGAAA
1419▸ A  L  P  S  L  T  N  P  P  S  L  S  P  I  R  R  K  G  K  E  K
4482 GAACCAGGAGAACAAGCATCTGTACCGTTGAGTCCCAAGAAAGGCAGTGAGGCCAGTGCAGCT
1440▸ E  P  G  E  Q  A  S  V  P  L  S  P  K  K  G  S  E  A  S  A  A
4545 TCCAGACAATCTGATACCTCAGGTCCTGTTACAACAAGTAAATCCTCATCACTGGGGAGTTTC
1461▸ S  R  Q  S  D  T  S  G  P  V  T  T  S  K  S  S  S  L  G  S  F
4608 TATCATCTTCCTTCATACCTCAAACTGCATGATGTCCTGAAAGCTACACACGCTAACTACAAG
1482▸ Y  H  L  P  S  Y  L  K  L  H  D  V  L  K  A  T  H  A  N  Y  K
4671 GTCACGCTGGATCTTCAGAACAGCACGGAAAAGTTTGGAGGGTTTCTCCGCTCAGCCTTGGAT
1503▸ V  T  L  D  L  Q  N  S  T  E  K  F  G  G  F  L  R  S  A  L  D
4734 GTTCTTTCTCAGATACTAGAGCTGGCCACACTGCAGGACATTGGGAAGTGTGTTGAAGAGATC
1524▸ V  L  S  Q  I  L  E  L  A  T  L  Q  D  I  G  K  C  V  E  E  I
4797 CTAGGATACCTGAAATCCTGCTTTTCTAGAGAACCAATGATGGCAACTGTTTGTGTTCAACAA
1545▸ L  G  Y  L  K  S  C  F  S  R  E  P  M  M  A  T  V  C  V  Q  Q
4860 TTGTTGAAGACTCTCTTTGGCACAAACTTGGCCTCCCAGTTTGATGGCTTATCTTCCAACCCC
1566▸ L  L  K  T  L  F  G  T  N  L  A  S  Q  F  D  G  L  S  S  N  P
4923 AGCAAGTCACAAGGCCGAGCACAGCGCCTTGGCTCCTCCAGTGTGAGGCCAGGCTTGTACCAC
1587▸ S  K  S  Q  G  R  A  Q  R  L  G  S  S  S  V  R  P  G  L  Y  H
4986 TACTGCTTCATGGCCCCGTACACCCACTTCACCCAGGCCCTCGCTGACGCCAGCCTGAGGAAC
1608▸ Y  C  F  M  A  P  Y  T  H  F  T  Q  A  L  A  D  A  S  L  R  N
5049 ATGGTGCAGGCGGAGCAGGAGAACGACACCTCGGGATGGTTTGATGTCCTCCAGAAAGTGTCT
1629▸ M  V  Q  A  E  Q  E  N  D  T  S  G  W  F  D  V  L  Q  K  V  S
5112 ACCCAGTTGAAGACAAACCTCACGAGTGTCACAAAGAACCGTGCAGATAAGAATGCTATTCAT
1650▸ T  Q  L  K  T  N  L  T  S  V  T  K  N  R  A  D  K  N  A  I  H
5175 AATCACATTCGTTTGTTTGAACCTCTTGTTATAAAGGCTTTAAAACAGTACACGACTACAACA
1671▸ N  H  I  R  L  F  E  P  L  V  I  K  A  L  K  Q  Y  T  T  T  T
5238 TGTGTGCAGTTACAGAAGCAGGTTTTAGATTTGCTGGCGCAGCTGGTTCAGTTACGGGTTAAT
1692▸ C  V  Q  L  Q  K  Q  V  L  D  L  L  A  Q  L  V  Q  L  R  V  N
5301 TACTGTCTTCTGGATTCAGATCAGGTGTTTATTGGCTTTGTATTGAAACAGTTTGAATACATT
1713▸ Y  C  L  L  D  S  D  Q  V  F  I  G  F  V  L  K  Q  F  E  Y  I
5364 GAAGTGGGCCAGTTCAGGGAATCAGAGGCAATCATTCCAAACATCTTTTTCTTCTTGGTATTA
1734▸ E  V  G  Q  F  R  E  S  E  A  I  I  P  N  I  F  F  F  L  V  L
5427 CTATCTTATGAACGCTATCATTCAAAACAGATCATTGGAATCCCTAAAATCATTCAGCTCTGT
1755▸ L  S  Y  E  R  Y  H  S  K  Q  I  I  G  I  P  K  I  I  Q  L  C
5490 GATGGCATCATGGCCAGTGGAAGGAAGGCTGTGACACATGCCATACCGGCTCTGCAGCCCATA
1776▸ D  G  I  M  A  S  G  R  K  A  V  T  H  A  I  P  A  L  Q  P  I
5553 GTCCACGACCTCTTTGTATTAAGAGGAACAAATAAAGCTGATGCAGGAAAAGAGCTTGAAACC
1797▸ V  H  D  L  F  V  L  R  G  T  N  K  A  D  A  G  K  E  L  E  T
5616 CAAAAAGAGGTGGTGGTGTCAATGTTACTGAGACTCATCCAGTACCATCAGGTGTTGGAGATG
1818▸ Q  K  E  V  V  V  S  M  L  L  R  L  I  Q  Y  H  Q  V  L  E  M
5679 TTCATTCTTGTCCTGCAGCAGTGCCACAAGGAGAATGAAGACAAGTGGAAGCGACTGTCTCGA
1839▸ F  I  L  V  L  Q  Q  C  H  K  E  N  E  D  K  W  K  R  L  S  R
5742 CAGATAGCTGACATCATCCTCCCAATGTTAGCCAAACAGCAGATGCACATTGACTCTCATGAA
1860▸ Q  I  A  D  I  I  L  P  M  L  A  K  Q  Q  M  H  I  D  S  H  E
                                                         SalI
5805 GCCCTTGGAGTGTTAAATACATTATTCGAAATTTTGGCCCCTTCCTCCCTCCGTCCGGTCGAC
1881▸ A  L  G  V  L  N  T  L  F  E  I  L  A  P  S  S  L  R  P  V  D
5868 ATGCTTTTACGGAGTATGTTCGTCACTCCAAACACAATGGCGTCCGTGAGCACTGTTCAACTG
1902▸ M  L  L  R  S  M  F  V  T  P  N  T  M  A  S  V  S  T  V  Q  L
```

Htt

5931 TGGATTTCGGGAATCCTGGCCATTTTGAGGGTTCTGATTTCCCAGTCAACTGAAGATATTGTT
1923▸ W I S G I L A I L R V L I S Q S T E D I V
5994 CTTTCTCGTATTCAGGAACTCTCCTTCTCTCCGTATTTAATCTCCTGCACAGTAATTAATAGG
1944▸ L S R I Q E L S F S P Y L I S C T V I N R
6057 TTAAGAGATGGGGACAGTACTTCAACGCTAGAAGAACACAGTGAAGGGAAACAAATAAAGAAT
1965▸ L R D G D S T S T L E E H S E G K Q I K N
6120 TTGCCAGAAGAAACATTTTCAAGGTTTCTATTACAACTGGTTGGTATTCTTTTAGAAGACATT
1986▸ L P E E T F S R F L L Q L V G I L L E D I
6183 GTTACAAAACAGCTGAAGGTGGAAATGAGTGAGCAGCAACATACTTTCTATTGCCAGGAACTA
2007▸ V T K Q L K V E M S E Q Q H T F Y C Q E L
6246 GGCACACTGCTAATGTGTCTGATCCACATCTTCAAGTCTGGAATGTTCAGGAGAATCACAGCA
2028▸ G T L L M C L I H I F K S G M F R R I T A
6309 GCTGCCACTAGGCTGTTCCGCAGTGATGGCTGTGGCGGCAGTTTCTACACCCTGGACAGCTTG
2049▸ A A T R L F R S D G C G G S F Y T L D S L
6372 AACTTGCGGGCTCGTTCCATGATCACCACCCACCCGGCCCTGGTGCTGCTCTGGTGTCAGATA
2070▸ N L R A R S M I T T H P A L V L L W C Q I
6435 CTGCTGCTTGTCAACCACACCGACTACCGCTGGTGGGCAGAAGTGCAGCAGACCCCGAAAAGA
2091▸ L L L V N H T D Y R W W A E V Q Q T P K R
6498 CACAGTCTGTCCAGCACAAAGTTACTTAGTCCCCAGATGTCTGGAGAAGAGGAGGATTCTGAC
2112▸ H S L S S T K L L S P Q M S G E E E D S D
6561 TTGGCAGCCAAACTTGGAATGTGCAATAGAGAAATAGTACGAAGAGGGGCTCTCATTCTCTTC
2133▸ L A A K L G M C N R E I V R R G A L I L F
6624 TGTGATTATGTCTGTCAGAACCTCCATGACTCCGAGCACTTAACGTGGCTCATTGTAAATCAC
2154▸ C D Y V C Q N L H D S E H L T W L I V N H
BglII
6687 ATTCAAGATCTGATCAGCCTTTCCCACGAGCCTCCAGTACAGGACTTCATCAGTGCCGTTCAT
2175▸ I Q D L I S L S H E P P V Q D F I S A V H
6750 CGGAACTCTGCTGCCAGCGGCCTGTTCATCCAGGCAATTCAGTCTCGTTGTGAAAACCTTTCA
2196▸ R N S A A S G L F I Q A I Q S R C E N L S
6813 ACTCCAACCATGCTGAAGAAAACTCTTCAGTGCTTGGAGGGGATTCATCTCAGCCAGTCGGGA
2217▸ T P T M L K K T L Q C L E G I H L S Q S G
6876 GCTGTGCTCACGCTGTATGTGGACAGGCTTCTGTGCACCCCTTTCCGTGTGCTGGCTCGCATG
2238▸ A V L T L Y V D R L L C T P F R V L A R M Htt
6939 GTGGACATCCTTGCTTGTCGCCGGGTAGAAATGCTTCTGGCTGCAAATTTACAGAGCAGCATG
2259▸ V D I L A C R R V E M L L A A N L Q S S M
7002 GCCCAGTTGCCAATGGAAGAACTCAACAGAATCCAGGAATACCTTCAGAGCAGCGGGCTCGCT
2280▸ A Q L P M E E L N R I Q E Y L Q S S G L A
7065 CAGAGACACCAAAGGCTCTATTCCCTGCTGGACAGGTTTCGTCTCTCCACCATGCAAGACTCA
2301▸ Q R H Q R L Y S L L D R F R L S T M Q D S
7128 CTTAGTCCCTCTCCTCCAGTCTCTTCCCACCCGCTGGACGGGGATGGGCACGTGTCACTGGAA
2322▸ L S P S P P V S S H P L D G D G H V S L E
7191 ACAGTGAGTCCCGACAAAGACTGGTACGTTCATCTTGTCAAATCCCAGTGTTGGACCAGGTCA
2343▸ T V S P D K D W Y V H L V K S Q C W T R S
7254 GATTCTGCACTGCTGGAAGGTGCAGAGCTGGTGAATCGGATTCCTGCTGAAGATATGAATGCC
2364▸ D S A L L E G A E L V N R I P A E D M N A
7317 TTCATGATGAACTCGGAGTTCAACCTAAGCCTGCTAGCTCCATGCTTAAGCCTAGGGATGAGT
2385▸ F M M N S E F N L S L L A P C L S L G M S
7380 GAAATTTCTGGTGGCCAGAAGAGTGCCCTTTTTGAAGCAGCCCGTGAGGTGACTCTGGCCCGT
2406▸ E I S G G Q K S A L F E A A R E V T L A R
7443 GTGAGCGGCACCGTGCAGCAGCTCCCTGCTGTCCATCATGTCTTCCAGCCCGAGCTGCCTGCA
2427▸ V S G T V Q Q L P A V H H V F Q P E L P A
7506 GAGCCGGCGGCCTACTGGAGCAAGTTGAATGATCTGTTTGGGGATGCTGCACTGTATCAGTCC
2448▸ E P A A Y W S K L N D L F G D A A L Y Q S
7569 CTGCCCACTCTGGCCAGAGCACTGGCACAGTACCTGGTGGTGGTCTCCAAACTGCCCAGTCAT
2469▸ L P T L A R A L A Q Y L V V V S K L P S H
7632 TTGCACCTTCCTCCTGAGAAAGAGAAGGACATTGTGAAATTCGTGGTGGCAACCCTTGAGGCC
2490▸ L H L P P E K E K D I V K F V V A T L E A
7695 CTGTCCTGGCATTTGATCCATGAGCAGATCCCGCTGAGTCTGGATCTCCAGGCAGGGCTGGAC
2511▸ L S W H L I H E Q I P L S L D L Q A G L D
7758 TGCTGCTGCCTGGCCCTGCAGCTGCCTGGCCTCTGGAGCGTGGTCTCCTCCACAGAGTTTGTG
2532▸ C C C L A L Q L P G L W S V V S S T E F V
7821 ACCCACGCCTGCTCCCTCATCTACTGTGTGCACTTCATCCTGGAGGCCGTTGCAGTGCAGCCT
2553▸ T H A C S L I Y C V H F I L E A V A V Q P

7884 GGAGAGCAGCTTCTTAGTCCAGAAAGAAGGACAAATACCCCAAAAGCCATCAGCGAGGAGGAG
2574▶ G E Q L L S P E R R T N T P K A I S E E E
7947 GAGGAAGTAGATCCAAACACACAGAATCCTAAGTATATCACTGCAGCCTGTGAGATGGTGGCA
2595▶ E E V D P N T Q N P K Y I T A A C E M V A
8010 GAAATGGTGGAGTCTCTGCAGTCGGTGTTGGCCTTGGGTCATAAAAGGAATAGCGGCGTGCCG
2616▶ E M V E S L Q S V L A L G H K R N S G V P
8073 GCGTTTCTCACGCCATTGCTAAGGAACATCATCATCAGCCTGGCCCGCCTGCCCCTTGTCAAC
2637▶ A F L T P L L R N I I I S L A R L P L V N

SpeI

8136 AGCTACACACGTGTGCCCCCACTAGTGTGGAAACTTGGATGGTCACCCAAACCAGGGGGGGAT
2658▶ S Y T R V P P L V W K L G W S P K P G G D
8199 TTTGGCACAGCATTCCCTGAGATCCCCGTGGAGTTCCTCCAGGAAAAGGAAGTCTTTAAGGAG
2679▶ F G T A F P E I P V E F L Q E K E V F K E
8262 TTCATCTACCGCATCAACACACTAGGCTGGACCAGTCGTACTCAGTTTGAAGAAACTTGGGCC
2700▶ F I Y R I N T L G W T S R T Q F E E T W A
8325 ACCCTCCTTGGTGTCCTGGTGACGCAGCCCCTCGTGATGGAGCAGGAGGAGAGCCCACCAGAA
2721▶ T L L G V L V T Q P L V M E Q E E S P P E
8388 GAAGACACAGAGAGGACCCAGATCAACGTCCTGGCCGTGCAGGCCATCACCTCACTGGTGCTC
2742▶ E D T E R T Q I N V L A V Q A I T S L V L
8451 AGTGCAATGACTGTGCCTGTGGCCGGCAACCCAGCTGTAAGCTGCTTGGAGCAGCAGCCCCGG
2763▶ S A M T V P V A G N P A V S C L E Q Q P R
8514 AACAAGCCTCTGAAAGCTCTCGACACCAGGTTTGGGAGGAAGCTGAGCATTATCAGAGGGATT
2784▶ N K P L K A L D T R F G R K L S I I R G I
8577 GTGGAGCAAGAGATTCAAGCAATGGTTTCAAAGAGAGAGAATATTGCCACCCATCATTTATAT
2805▶ V E Q E I Q A M V S K R E N I A T H H L Y
8640 CAGGCATGGGACCCTGTCCCTTCTCTGTCTCCGGCTACTACAGGTGCCCTCATCAGCCACGAG
2826▶ Q A W D P V P S L S P A T T G A L I S H E
8703 AAGCTGCTGCTACAGATCAACCCCGAGCGGGAGCTGGGGAGCATGAGCTACAAACTCGGCCAG
2847▶ K L L L Q I N P E R E L G S M S Y K L G Q
8766 GTGTCCATACACTCCGTGTGGCTGGGGAACAGCATCACACCCCTGAGGGAGGAGGAATGGGAC
2868▶ V S I H S V W L G N S I T P L R E E E W D
8829 GAGGAAGAGGAGGAGGAGGCCGACGCCCCTGCACCTTCGTCACCACCCACGTCTCCAGTCAAC
2889▶ E E E E E E A D A P A P S S P P T S P V N
8892 TCCAGGAAACACCGGGCTGGAGTTGACATCCACTCCTGTTCGCAGTTTTTGCTTGAGTTGTAT
2910▶ S R K H R A G V D I H S C S Q F L L E L Y
8955 AGCCGCTGGATACTGCCGTCCAGCTCAGCCAGGAGGACCCCGGCCATCCTGATCAGTGAGGTG
2931▶ S R W I L P S S S A R R T P A I L I S E V
9018 GTCAGATCCCTTCTAGTGGTCTCAGACTTGTTCACCGAGCGCAACCAGTTTGAGCTGATGTAT
2952▶ V R S L L V V S D L F T E R N Q F E L M Y
9081 GTGACGCTGACAGAACTGCGAAGGGTGCACCCTTCAGAAGACGAGATCCTCGCTCAGTACCTG
2973▶ V T L T E L R R V H P S E D E I L A Q Y L

XhoI

9144 GTGCCTGCCACCTGCAAGGCAGCTGCCGTCCTTGGGATGGACAAGGCCGTGGCGGAGCCTGTC
2994▶ V P A T C K A A A V L G M D K A V A E P V
9207 TCGAGGCTGCTGGAGAGCACGCTCAGGAGCAGCCACCTGCCCAGCAGGGTTGGAGCCCTGCAC
3015▶ S R L L E S T L R S S H L P S R V G A L H
9270 GGCGTCCTCTATGTGCTGGAGTGCGACCTGCTGGACGACACTGCCAAGCAGCTCATCCCGGTC
3036▶ G V L Y V L E C D L L D D T A K Q L I P V
9333 ATCAGCGACTATCTCCTCTCCAACCTGAAAGGGATCGCCCACTGCGTGAACATTCACAGCCAG
3057▶ I S D Y L L S N L K G I A H C V N I H S Q
9396 CAGCACGTACTGGTCATGTGTGCCACTGCGTTTTACCTCATTGAGAACTATCCTCTGGACGTA
3078▶ Q H V L V M C A T A F Y L I E N Y P L D V
9459 GGGCCGGAATTTTCAGCATCAATAATACAGATGTGTGGGGTGATGCTGTCTGGAAGTGAGGAG
3099▶ G P E F S A S I I Q M C G V M L S G S E E
9522 TCCACCCCCTCCATCATTTACCACTGTGCCCTCAGAGGCCTGGAGCGCCTCCTGCTCTCTGAG
3120▶ S T P S I I Y H C A L R G L E R L L L S E
9585 CAGCTCTCCCGCCTGGATGCAGAATCGCTGGTCAAGCTGAGTGTGGACAGAGTGAACGTGCAC
3141▶ Q L S R L D A E S L V K L S V D R V N V H
9648 AGCCCGCACCGGGCCATGGCGGCTCTGGGCCTGATGCTCACCTGCATGTATACAGGAAAGGAG
3162▶ S P H R A M A A L G L M L T C M Y T G K E
9711 AAAGTCAGTCCGGGTAGAACTTCAGACCCTAATCCTGCAGCCCCCGACAGCGAGTCAGTGATT
3183▶ K V S P G R T S D P N P A A P D S E S V I
9774 GTTGCTATGGAGCGGGTATCTGTTCTTTTTGATAGGATCAGGAAAGGCTTTCCTTGTGAAGCC
3204▶ V A M F R V S V I F D R I R K G F P C F A

Htt

```
9837  AGAGTGGTGGCCAGGATTCTGCCCCAGTTCCTAGACGACTTCTTCCCACCCCAGGACATCATG
3225▸ R  V  V  A  R  I  L  P  Q  F  L  D  D  F  F  P  P  Q  D  I  M
9900  AACAAAGTCATCGGAGAGTTTCTGTCCAACCAGCAGCCATACCCCCAGTTCATGGCCACCGTG
3246▸ N  K  V  I  G  E  F  L  S  N  Q  Q  P  Y  P  Q  F  M  A  T  V
9963  GTGTATAAGGTGTTTCAGACTCTGCACAGCACCGGGCAGTCGTCCATGGTCCGGGACTGGGTC
3267▸ V  Y  K  V  F  Q  T  L  H  S  T  G  Q  S  S  M  V  R  D  W  V      Htt
10026 ATGCTGTCCCTCTCCAACTTCACGCAGAGGGCACCAGTCGCCATGGCCACGTGGAGCCTCTCC
3288▸ M  L  S  L  S  N  F  T  Q  R  A  P  V  A  M  A  T  W  S  L  S
10089 TGCTTCTTTGTCAGCGCGTCCACCAGCCCGTGGGTCGCGGCGATCCTCCCACATGTCATCAGC
3309▸ C  F  F  V  S  A  S  T  S  P  W  V  A  A  I  L  P  H  V  I  S
10152 AGGATGGGCAAGCTGGAGCAGGTGGACGTGAACCTTTTCTGCCTGGTCGCCACAGACTTCTAC
3330▸ R  M  G  K  L  E  Q  V  D  V  N  L  F  C  L  V  A  T  D  F  Y
10215 AGACACCAGATAGAGGAGGAGCTGGACCGCAGGGCCTTCCAGTCTGTGCTTGAGGTGGTTGCA
3351▸ R  H  Q  I  E  E  E  L  D  R  R  A  F  Q  S  V  L  E  V  V  A
10278 GCCCCAGGAAGCCCATATCACCGGCTGCTGACTTGTTTACGAAATGTCCACAAGGTCACCACC
3372▸ A  P  G  S  P  Y  H  R  L  L  T  C  L  R  N  V  H  K  V  T  T
         EcoRI  SmaI             HA tag             NdeI         TC tag
10341 TGCGAATTCCCCGGGTTTTACCCCTATGATGTGCCAGACTACGCCCATATGGGGGGGTTCTTG
3393▸ C  E  F  P  G  F  Y  P  Y  D  V  P  D  Y  A  H  M  G  G  F  L
                                   FseI       FspI      SphI   SnaBI     attL2
10404 AATTGCTGTCCTGGCTGCTGCATGGAACCTGGCCGGCCATGCGCATAAGCATGCTACGTACACCCAG
3414▸ N  C  C  P  G  C  C  M  E  P  G  R  P  C  A
10471 CTTTCTTGTACaaagttggcattataagaaagcattgcttatcaatttgttgcaacgaacaggtcactatcagt
10545 caaaataaaatcattatttgccatccagctgcagctctggcccgtgtctcaaaatctctgatgttacattgcac
10619 aagataaaaatatatcatcatgaacaataaaactgtctgcttacataaacagtaatacaaggggtgttatgagc
10693 catattcaacgggaaacgtcgaggccgcgattaaattccaacatggatgctgatttatatgggtataaatgggc
10767 tcgcgataatgtcgggcaatcaggtgcgacaatctatcgcttgtatgggaagcccgatgcgccagagttgtttc
10841 tgaaacatggcaaaggtagcgttgccaatgatgttacagatgagatggtcagactaaactggctgacggaattt
10915 atgcctcttccgaccatcaagcattttatccgtactcctgatgatgcatggttactcaccactgcgatccccgg
10989 aaaaacagcattccaggtattagaagaatatcctgattcaggtgaaaatattgttgatgcgctggcagtgttcc
11063 tgcgccggttgcattcgattcctgtttgtaattgtccttttaacagcgatcgcgtatttcgtctcgctcaggcg
11137 caatcacgaatgaataacggtttggttgatgcgagtgattttgatgacgagcgtaatggctggcctgttgaaca
11211 agtctggaaagaaatgcataaacttttgccattctcaccggattcagtcgtcactcatggtgatttctcacttg
11285 ataaccttatttttgacgaggggaaattaataggttgtattgatgttggacgagtcggaatcgcagaccgatac
11359 caggatcttgccatcctatggaactgcctcggtgagttttctccttcattacagaaacggctttttcaaaaata
11433 tggtattgataatcctgatatgaataaattgcagtttcatttgatgctcgatgagtttttctaatcagaattgg
11507 ttaattggttgtaacactggcagagcattacgctgacttgacgggacggcgcaagctcatgaccaaaatccctt
11581 aacgtgagttttcgttccactgagcgtcagaccccgtagaaaagatcaaaggatcttcttgagatcctttttt
11655 ctgcgcgtaatctgctgcttgcaaacaaaaaaaccaccgctaccagcggtggtttgtttgccggatcaagagct
11729 accaactctttttccgaaggtaactggcttcagcagagcgcagataccaaatactgtccttctagtgtagccgt
11803 agttaggccaccacttcaagaactctgtagcaccgcctacatacctcgctctgctaatcctgttaccagtggct
11877 gctgccagtggcgataagtcgtgtcttaccgggttggactcaagacgatagttaccggataaggcgcagcggtc
11951 gggctgaacggggggttcgtgcacacagcccagcttggagcgaacgacctacaccgaactgagatacctacagc
12025 gtgagctatgagaaagcgccacgcttcccgaagggagaaaggcggacaggtatccggtaagcggcagggtcgga
12099 acaggagagcgcacgagggagcttccagggggaaacgcctggtatctttatagtcctgtcgggtttcgccacct
12173 ctgacttgagcgtcgatttttgtgatgctcgtcaggggggcggagcctatggaaaaacgccagcaacgcggcct
12247 ttttacggttcctggccttttgctggccttttgctcacatgttctttcctgcgttatcccctgattctgtggat
12321 aaccgtattaccgctagccaggaagagtttgtagaaacgcaaaaaggccatccgtcaggatggccttctgctta
12395 gtttgatgcctggcagtttatggcgggcgtcctgcccgccaccctccgggccgttgcttcacaacgttcaaatc
12469 cgctcccggcggatttgtcctactcaggagagcgttcaccgacaaacaacagataaaacgaaaggcccagtctt
                                                                junction marker
12543 ccgactgagcctttcgttttatttgatgcctggcagttccctactctc
```

Full name : pARIS-htt-N[His-Cherry]Q100-C[HA-TC]
Used name : pARIS-mCherry-httQ100
vector : pENTRY

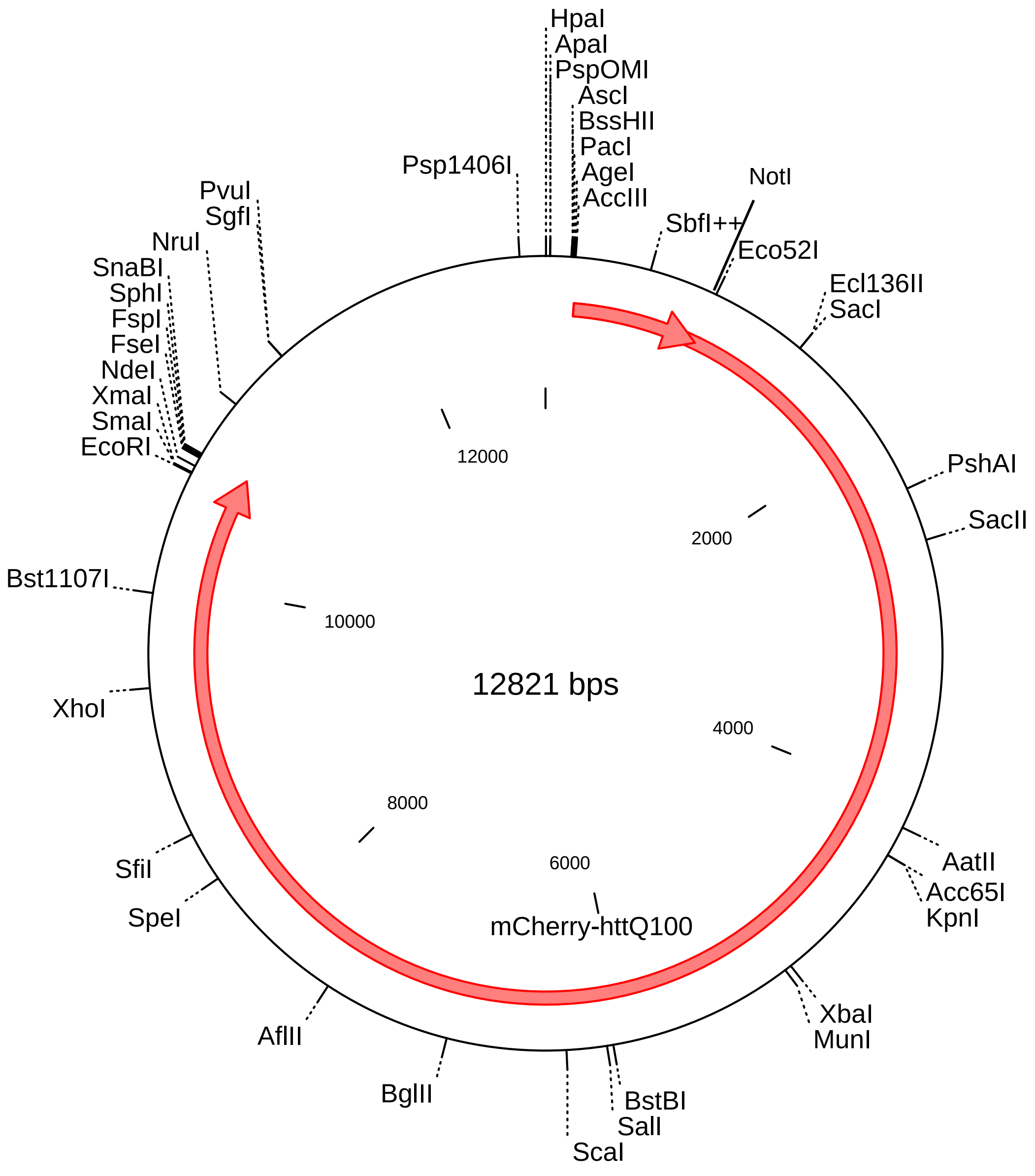

gene : mCherry-httQ100
- start : 165
- end 10682

Molecule: pARIS-mCherry-httQ100, 12821 bps DNA Circular
Description:
File Name: pARIS-mCherry-httQ100.cm5, dated 23 Apr 2010
Printed: List of Sites in bps, sorted by Enzyme name.
Filter ON: cut N <= 1

	#sites	----- Bp position of recognition site -----
AatII	1	4131
Acc65I	1	4292
AccIII	1	155
AflII	1	7592
AgeI	1	149
ApaI	1	24
AscI	1	133
BglII	1	6923
BssHII	1	134
Bst1107I	1	9926
BstBI	1	6060
Ecl136II	1	1419
Eco52I	1	907
EcoRI	1	10575
FseI	1	10665
FspI	1	10674
HpaI	1	3
KpnI	1	4292
MunI	1	5088
NdeI	1	10617
NotI	1	906
NruI	1	10998
PacI	1	141
PshAI	1	2333
Psp1406I	1	12688
PspOMI	1	24
PvuI	1	11340
SacI	1	1419
SacII	1	2614
SalI	1	6093
SbfI	1	548
ScaI	1	6303
SfiI	1	8651
SgfI	1	11339
SgrAI	1	878
SmaI	1	10581
SnaBI	1	10689
SpeI	1	8387
SphI	1	10683
XbaI	1	5052
XhoI	1	9437
XmaI	1	10581
